# Experimental and Computational Studies on the Interaction of a Dansyl-Based Fluorescent Schiff Base Ligand with Cu^2+^ Ions and CuO NPs

**DOI:** 10.3390/ijms231911565

**Published:** 2022-09-30

**Authors:** Jesús Sanmartín-Matalobos, Pilar Bermejo-Barrera, Ignacio Pérez-Juste, Matilde Fondo, Ana M. García-Deibe, Yeneva Alves-Iglesias

**Affiliations:** 1 Coordination and Supramolecular Chemistry Group (SupraMetal), Institute of Materials (iMATUS), Department of Inorganic Chemistry, Faculty of Chemistry, Universidade de Santiago de Compostela, Avenida das Ciencias s/n, 15782 Santiago de Compostela, Spain; 2 Trace Element, Speciation and Spectroscopy Group (GETEE), Institute of Materials (iMATUS), Department of Analytical Chemistry, Nutrition and Bromatology, Faculty of Chemistry, Universidade de Santiago de Compostela, Avenida das Ciencias s/n, 15782 Santiago de Compostela, Spain; 3 Departamento de Química Física, Facultad de Química, Edificio de Ciencias Experimentales, Universidade de Vigo, 36310 Vigo, Spain

**Keywords:** CuO nanoparticles, nanomaterials, detection, luminescence, Schiff base, fluorescent chemosensor, fluorescence quenching

## Abstract

We studied the interaction of Cu^2+^ ions and CuO nanoparticles with the fluorescent Schiff base ligand H_3_L, which derives from the condensation of 4-formyl-3-hydroxybenzoic acid with *N*-(2-aminobenzyl)-5-(dimethylamino)naphthalene-1-sulfonamide (DsA). A detailed assignment of the most significant bands of the electronic and infrared spectra of H_3_L and DsA was performed using DFT methods, based on both crystal structures. The affinity of H_3_L to react with Cu^2+^ ions in solution (K_B_ = 9.01 10^3^ L mol^−1^) is similar to that found for the Cu^2+^ ions present on the surface of CuO NPs (K_B_ = 9.84 10^3^ L mol^−1^). Fluorescence spectroscopic measurements suggest five binding sites for H_3_L on the surface of the CuO NPs used. The µ-XRF analysis indicates that a polycrystalline sample of CuO-H_3_L NPs contains 15:1 Cu:S molar ratio (CuO:H_3_L). ATR-FTIR spectroscopy, supported by DFT calculations, showed that the HL^2−^ (as a phenolate and sulfonamide anion) is coordinated to superficial Cu^2+^ ions of the CuO NPs through their azomethine, sulphonamide, and phenolic groups. A solution of H_3_L (126 ppb) shows sensitive responses to CuO NPs, with a limit of detection (LOD) of 330 ppb. The working range for detection of CuO NPs with [H_3_L] = 126 ppb was 1.1–9.5 ppm. Common metal ions in water, such as Na^+^, K^+^, Mg^2+^, Ca^2+^, Fe^3+^, and Al^3+^ species, do not interfere significantly with the detection of CuO NPs.

## 1. Introduction

The unique properties of metal and metal oxide nanomaterials have led to their widespread use in daily life [1,2,3,4,5], which has the clear negative effect of an increasing release of these nanomaterials into the environment [6,7,8,9,10]. Particularly, CuO NPs are manufactured on a large scale for their varied industrial and domestic applications, as they are generally considered to be non-toxic. Thus, one of the common usages of CuO NPs is in water treatment, as an effective adsorbent of several pollutants [4]. However, some concern is arising about the possible adverse health and environmental effects of their release since several studies claim that CuO NPs could have negative impacts on different living organisms [11,12,13].

Therefore, the development and improvement of methods for the detection of CuO NPs is a growing field of study. Thus, Zhao et al. used EDS analysis to quantify the presence of copper on the root surface of an aquatic plant, after CuO NP exposure [14]. ICP-OES was used by Peng et al. to measure the copper content in root of rice after adding CuO NPs to the soil [15], while Navratilova et al. applied the single particle ICP-MS method for the detection of CuO NPs in colloidal extracts from natural soil samples [16]. In this sense, our research group has also used a pyrrole-based [17], as well as phenol-based Schiff base ligands [18] as fluorescent sensors for the detection of CuO NPs (LOD = 13.83 and 9.80 μgL^−1^, respectively). These two *N*-tosyl imines have a moderate fluorescence emission, which drastically decreases by interaction with copper ions present on the surface of the CuO NPs. In the present study, with the aim of avoiding the disadvantages of using weakly fluorescent ligands, we designed a new sensor with higher fluorescence emission intensity providing potential N and O donor atoms, which have demonstrated to be suitable for coordinating those superficial copper ions present on the surface of the CuO nanoparticles [17,18].

**Scheme 1:**
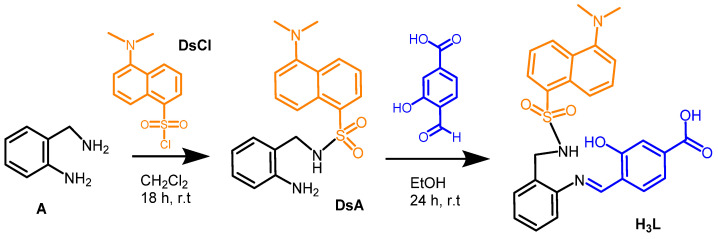
Schematic representation of the synthesis of H_3_L in two steps: (i) selective dansylation of a primary diamine and (ii) aldiminic condensation of DsA with 4-formyl-3-hydroxybenzoic acid.

We investigated the ability of the dansyl-based fluorescent Schiff base ligand H_3_L for detecting CuO NPs in ethanol-water solutions, with short response time and sensing at basic pH. H_3_L can be easily synthesized (Scheme 1) from the condensation of 4-formyl-3-hydroxybenzoic acid with *N*-(2-aminobenzyl)-5-(dimethylamino)naphthalene-1-sulfonamide (DsA). This dansyl-derived amine had been previously obtained by selective reaction of 2-(aminomethyl)aniline with dansyl chloride [19]. H_3_L displays an *N,N,O*-binding domain suitable to interact with Cu^2+^ ions, either in solution, or as constituents of a solid matrix as CuO NPs. In this case, additional O atoms belonging to carboxylate and sulfonamide groups can also be involved in the interaction.

Since the knowledge of the surface coordination chemistry of NPs [20,21,22,23] is advantageous for the development of chemical sensors for them, we also present here some experimental and computational studies on the interactions of H_3_L with CuO NPs. SEM-EDX, µ-XRF, ATR-IR, and DFT were used to investigate the interaction of H_3_L with the surface of CuO NPs. With the aim of assisting in the interpretation of the results obtained from the study of the interaction between H_3_L and CuO NPs, the copper(II) complex, Cu_2_L_2_(H_2_O)_4_, was synthesized and studied by ATR-IR, UV-Vis, and fluorescence spectroscopies. DFT calculations have been used to predict the effect of the symmetry of DsA, H_3_L, and Cu_2_L_2_(H_2_O)_4_ on their vibrational modes and electronic properties. Binding constants have been also determined since these constants characterize the affinity of H_3_L for both NMs and ions. Changes in the fluorescence emission spectra of H_3_L upon increasing addition of CuO NPs have been investigated as well, while taking into consideration possible interferences from some common metal ions in water.

## 2. Results and Discussion

With the aim of investigating the ability of a dansyl-based fluorescent Schiff base ligand for detecting copper oxide nanomaterials in ethanol-water samples, we designed the easily synthesized H_3_L (Scheme 1). An ethanol solution of H_3_L at neutral pH emitted a maximal green fluorescence at about 524 nm, when it was exposed to radiation with a wavelength of 400 nm. This fluorescent behavior was assigned to the dansyl moiety, which is well known for a typical charge-transfer band between the donor dimethylamino group and the sulfonyl group [24].

H_3_L can be obtained in a quick two-step synthesis (Scheme 1). In the first step, 2-(aminomethyl)aniline selectively reacts, through the aminomethyl group, with dansyl chloride to yield *N*-(2-aminobenzyl)-5-(dimethylamino)naphthalene-1-sulfonamide (DsA). Subsequent nucleophilic addition of DsA to 4-formyl-3-hydroxybenzoic acid leads to the desired Schiff base ligand H_3_L [19]. Both reactions were performed at room temperature, in order to prevent the dansylation of both amino groups in the first step, as well as undesired chemical reactions of DsA in the second step. Spectroscopic characterization details of H_3_L and DsA, obtained as powdery solids, are shown in the experimental section. Figures S1 and S2 show the ^1^H NMR spectra of H_3_L and DsA (in dmso-d_6_), respectively. Single crystals of [H_4_L]Cl·0.92H_2_O were obtained by slow evaporation of an ethanol solution of DsA and 4-formyl-3-hydroxybenzoic acid, to which triethylammonium chloride ([HNEt_3_]^+^, pK_a_ = 10.75) had been added.

### 2.1. Crystal Structures for H_3_L and DsA

**Figure 1:**
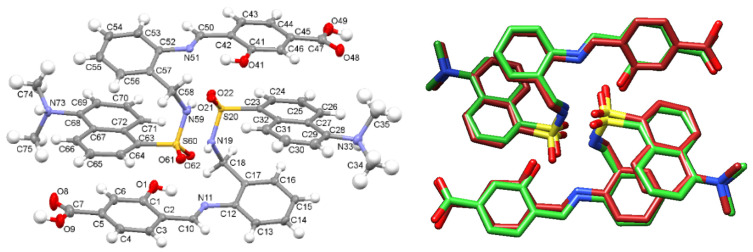
**(Left**): Ellipsoid view of the molecular structure of the two H_4_L^+^ cations present in the asymmetric unit of [H_4_L]Cl·0.92H_2_O crystals. (**Right**): Superimposition of the experimental structure and the theoretically calculated one (M062X/6-31G*) as sticks diagrams.

The molecular structure of two neighboring [H_4_L]^+^ cations is shown in Figure 1, as an ellipsoid diagram, which includes a labelling scheme equivalent to that used in the crystal structure of DsA (see below). Some significant crystal parameters and refinement data are summarized in Table S1. The asymmetric unit of [H_4_L]Cl·0.92H_2_O consists of two crystallographically independent cationic moieties of [H_4_L]^+^, showing their amine N atoms protonated (N33 and N73), two chloride counterions, and several water molecules with low occupation sites, which sum up to 0.92, per asymmetric unit. It must be noted that one of the chloride anions, as well as one of the dansyl residues (C63–C75) are disordered on two different occupation sites (0.832 and 0.168).

Geometric parameters (Tables S2 and S3) are within typical ranges observed for residues related to DsA [25,26,27,28] and 4-formyl-3-hydroxybenzoic acid [29,30,31]. Strong intramolecular H bonds (Table S4) O(1)-H(1)···N(11) and O(41)-H(41)···N(51), as well as π-stacking interactions stabilize the configuration of these two molecules. Mutual intermolecular bonds, N(19)-H(19)···O(61), and N(59)-H(59)···O(21), are responsible for connecting adjacent molecules, which as a result are forming H-bonded dimers shown in Figure 1. The crystal packing is reinforced through interactions between HMe_2_N^+^- and HOOC- groups with solvated water molecules, as well as with chloride counterions.

Since the experimental geometry available for the H_3_L ligand corresponds to that of two neighboring cationic units found in the [H_4_L]Cl·0.92 H_2_O crystal, these data were used to obtain the theoretical structure for a H-bonded dimer of H_3_L (Figure S3). This M062X/6-31G* optimized geometry shows an excellent agreement with the structure of the asymmetric unit of the [H_4_L]Cl·0.92H_2_O crystal (Figure 1, right), with differences between experimental and theoretical values for bond lengths and bond angles lower than 0.06 Å and 3.5^o^, respectively (Table S5).

**Figure 2:**
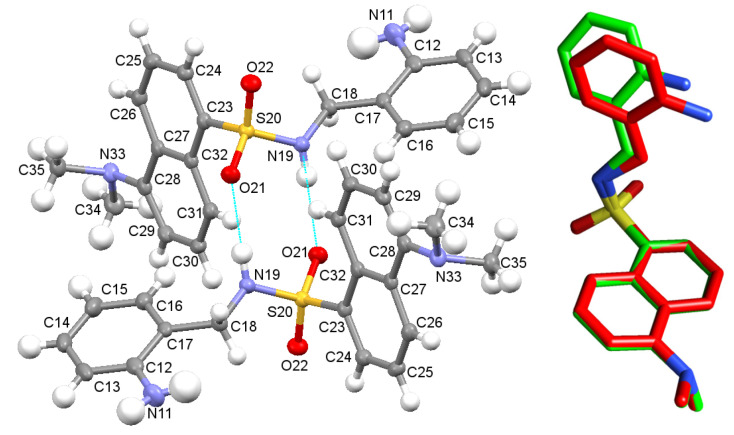
(**Left**): Ellipsoid diagram of the molecular structure found for DsA, including its labelling scheme. Two neighboring molecules are showing their mutual interaction. (**Right**): Superimposition of the stick diagrams of the experimental structure and the calculated one (M062X/6-31G*) for a single unit of DsA.

Moreover, we solved the crystal structure of the precursor DsA, which is shown in Figure 2. Details of the crystal data collection and structure refinement are also summarized in Table S1. Crystals of DsA consist of crystallographically independent molecules, whose main geometric parameters (Tables S2 and S3) are within the typical ranges observed for other dansyl derivatives [25,26,27,28]. Mutual intermolecular N(19)-H(19A)···O(21)^#3^ bonds allow connecting two contiguous molecules to form H-bonded dimers, as occurred for its derivative [H_4_L]Cl·0.92 H_2_O as well. Sulfonamide oxygen atoms of DsA are additionally connected to other adjacent molecules through the amino group N(11)-H(11B)···O(21)^#4^ (Table S6), as typically observed for dansyl [25] and tosyl [32] derivatives.

Taking this crystallographic information as a starting point, we also obtained the M062X/6-31G* optimized geometry for a single unit of DsA (Figure S4). The good agreement between the experimental structure and the calculated one is shown in Figure 2 (right), with differences for bond lengths and bond angles lower than 0.05 Å and 3.5^o^, respectively (Table S7). The theoretical conformational analysis of DsA supports that the preference for the experimentally crystal structure here presented is related to the stabilization facilitated by hydrogen bonds between adjacent molecules in the solid phase.

### 2.2. Electronic and Vibrational Spectra of H_3_L and DsA

DFT calculations at the M062X/6-31G* level of theory were used to assist in the correct assignment of bands in the electronic and vibrational spectra of H_3_L and DsA.

**Figure 3:**
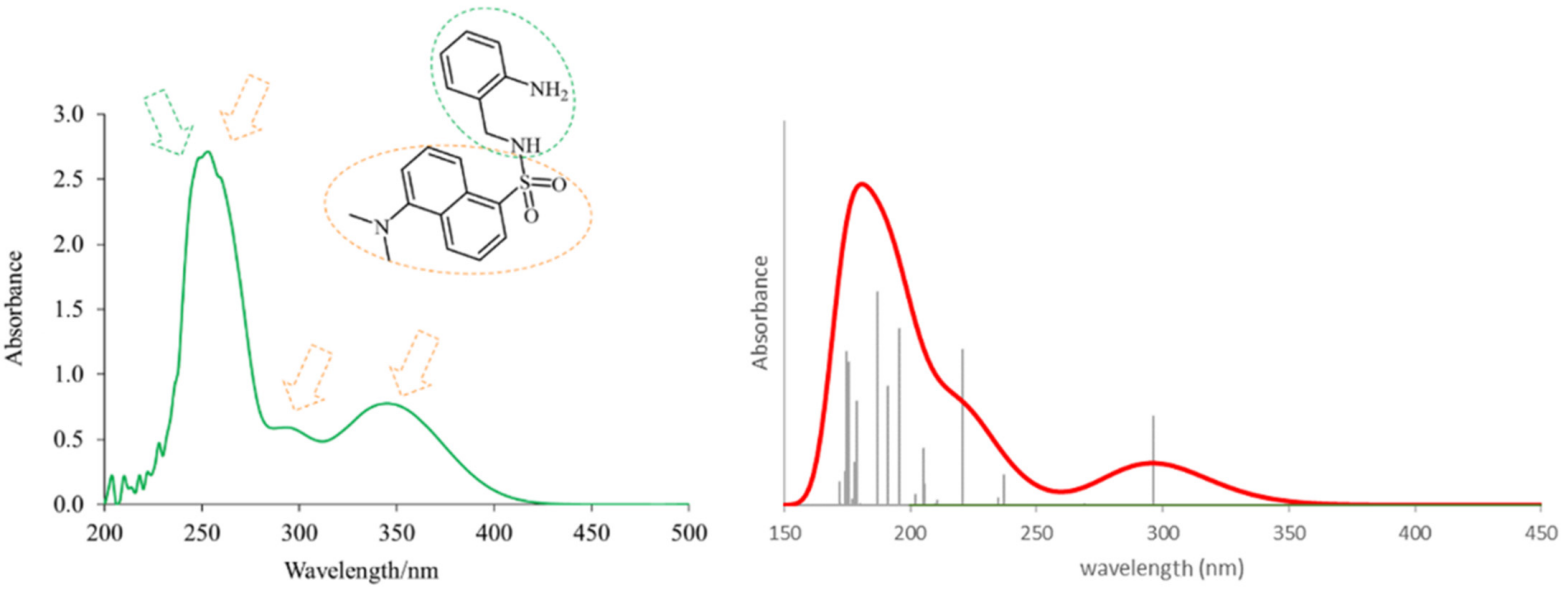
Experimental (**left**) and theoretical (**right**) profiles for the electronic spectrum of DsA. The signal assignment of the two moieties of the molecule has been included as a schematic. The orange arrows show the bands associated with the dansyl moiety, and the green arrow shows the band associated with the aniline moiety. Vertical lines indicate individual electronic transitions listed in Table 1.

The theoretical electronic spectrum of DsA (Figure 3) shows three dominant signals around 180, 220, and 295 nm that appear about 50–70 nm blue-shifted, with respect to the bands experimentally observed (252, 294, and 346 nm). The analysis of their natural transition orbitals (Table 1) indicates that the band at higher wavelengths mainly corresponds to a π-π* transition in the dansyl residue. The band at 220 nm corresponds to a markedly mixed n-π* and π-π* transition in the dansyl unit as well. Finally, among the several electronic transitions that contribute to the intense band at lower wavelengths, the most intense ones are two π-π* transitions located in the dansyl unit, and a π-π* transition located in the aniline moiety.

**Figure 4:**
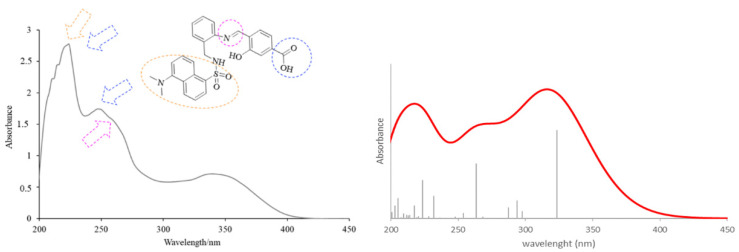
Experimental (**left**) and theoretical (**right**) profiles for the electronic spectrum of H_3_L. The signal assignment of the main groups of the molecule has been included as a scheme. The orange arrow shows the bands associated with the dansyl moiety. The blue arrows show the band associated with the carboxylic group and the pink arrow shows the band associated with the azomethine group. Vertical lines indicate individual electronic transitions listed in Table 1.

The theoretical electronic spectrum of H_3_L (Figure 4, right) also shows three dominant signals located around 323, 263, and 224 nm, which appear blue-shifted with respect to those experimentally observed (340, 248, and 224 nm) (Figure 4, left). The analysis of the molecular orbitals involved in each electronic transition of H_3_L, and the corresponding natural transition orbitals (Table 2), indicates that the bands at 323 and 263 nm correspond to π-π* transitions mainly centered in the quasi-planar and conjugated hydroxybenzoic/aniline region. The occupied orbital for the transition at 263 nm also shows a noticeable contribution from orbitals centered in the C=N group. The low wavelength maximum at 224 nm corresponds to a strongly mixed transition, which can also be mainly characterized as a π-π* transition centered in the orbitals of the π- stacked aromatic rings.

The assignment of the more significant bands of the infrared spectra of H_3_L (Table 3) and DsA (Table 4) has also been done on the basis of the calculated spectrum using DFT methods. The IR spectrum of H_3_L shows a characteristic new strong sharp band at about 1613 cm^−1^, which is attributable to the formation of the imino group (Table 3). This band evidences the condensation through the amino group of DsA with the 4-formyl-3-hydroxybenzoic acid. Furthermore, as a result of this condensation, the absence of the two bands at about 3470 and 3383 cm^−1^, attributable to ν_a_ NH_2_ and ν_s_ NH_2_ modes, respectively, is clear in the spectrum of DsA. Figures S5 and S6 show the experimental and calculated IR spectra of H_3_L and DsA, respectively. The characteristic O-H_(phenol)_ and N-H_(sulfonamide)_ stretches are observed as a single signal at 3283 cm^−1^ in the spectrum of H_3_L. Asymmetric and symmetric O=S=O stretches can also be clearly observed at about 1315 and 1140 cm^−1^, respectively, in the IR spectra of H_3_L and DsA. Tables S8 and S9 list a detailed assignment of experimental and theoretical vibrational frequencies of H_3_L and DsA, respectively.

### 2.3. Experimental and Theoretical Studies on the Copper(II) Complex

Cu_2_L_2_(H_2_O)_4_ was obtained at room temperature from the reaction between H_3_L and Cu(OAc)_2_, and alternatively, by the electrochemical oxidation of a copper anode immersed in a solution of H_3_L. It must be noted that the electrochemical procedure saves time and leads to higher yields (see experimental section). The copper(II) complex obtained from H_3_L has been investigated in solution by a combination of UV-Vis and fluorescence spectrometries. In the solid state, this complex has been investigated by elemental analysis and ATR-FTIR spectroscopy. Characterization details are shown in the experimental section. The results of the CHNS analyses showed that the copper(II) complex was obtained with a 1:1 metal:ligand stoichiometry. This finding is coherent with the formation of a sulfonamido-bridged dimeric complex, as those reported by us for complexes of related Schiff base ligands [17,33,34].

The IR spectrum of this copper(II) complex showed a broad band centered at about 3360 cm^−1^ attributable to ν(OH), which evidenced the formation of the hydrated complex (Figure S7). The slight shifts of ν(NC), ν_as_(SO_2_), and ν_s_(SO_2_) to lower wavenumbers (1–5 cm^−1^), as well as the absence of the band related to the phenolic COH bending (1412 cm^−1^), is a clear sign of the coordination of the Schiff base ligand through its O_phenol_, N_imine_, and N_sulfonamide_ donor atoms. The observation of two bands attributable to ν_as_(COO^−^) and ν_s_(COO^−^) at about 1574 and 1393 cm^−1^, respectively, instead of a single band at 1686 cm^−1^ indicates the deprotonation of the carboxylic group of the ligand in this complex. The net charge zero of the complex can be easily achieved, as the ligand units can adopt the zwitterionic form, in which the carboxylic groups are deprotonated, while the amine N atoms are protonated.

The electronic spectrum of the dimeric copper(II) complex (Figure S8) shows three dominant signals around 219, 244, and 336 nm appearing ca. 5 nm blue-shifted with respect to the free ligand (224, 248, and 340 nm). The metal complexation of H_3_L with Cu^2+^ is related to a new absorption band at about 410 nm, and which has been assigned to a ligand-to-metal charge transfer (LMTC).

**Figure 5:**
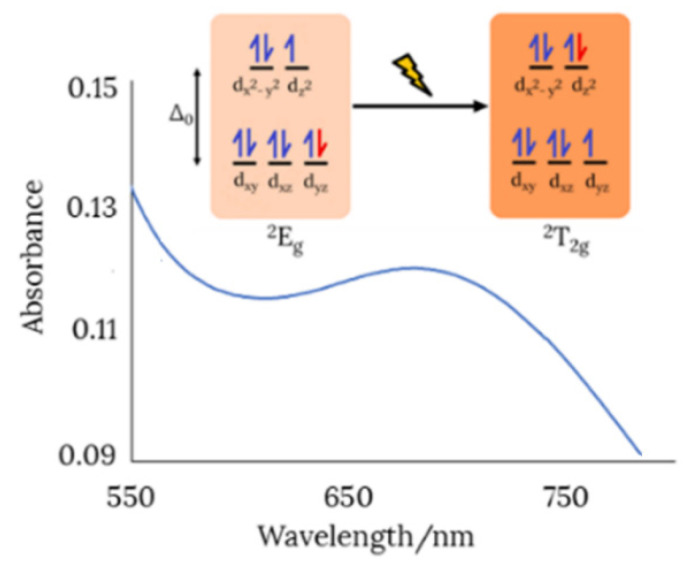
View of the *d-d* transition in the UV-Vis spectrum of the dimeric copper(II) complex (in ethanol).

The observation of a new absorption band at about 680 nm attributable to a *d*-*d* transition (^2^E_g_ ← ^2^T_2g_) in the UV-Vis spectrum of the dinuclear copper(II) complex (Figure 5), is a sign of six-coordination [35]. Therefore, two water molecules must complete the coordination sphere around each metal ion, occupying the vacant positions left by the dianionic Schiff base ligand.

**Figure 6:**
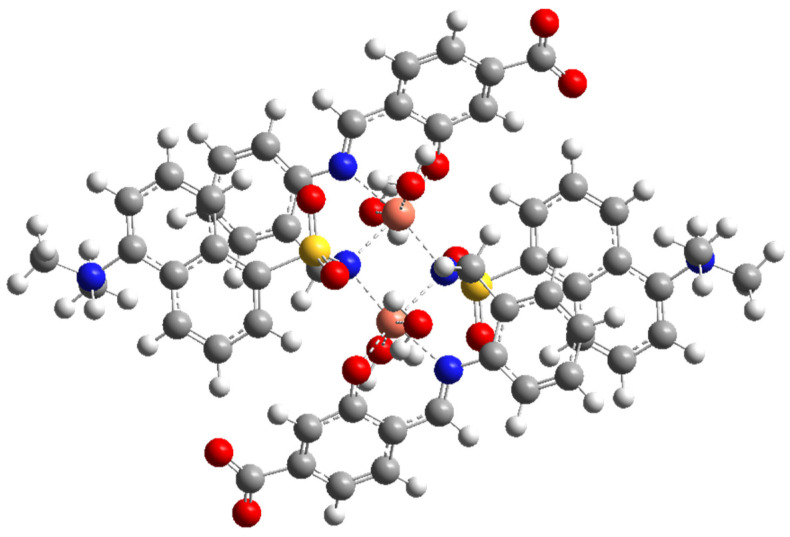

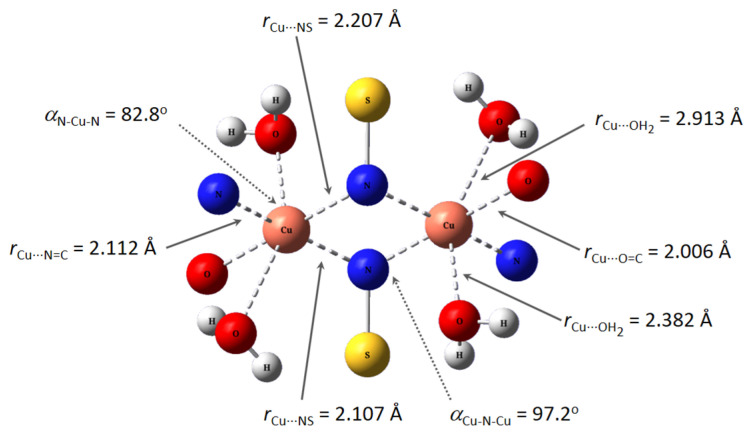
(**Top**): M062X/6-31G* optimized structure of Cu_2_(HL)_2_(H_2_O)_4_. (**Bottom**): Some bond distances and angles in the coordination environment of the copper ions.

The geometrical structure of the copper(II) complex has been analyzed by means of theoretical methods. Based on experimental results (CHNS analyses, infrared, and electronic spectra) and previous studies [17,33,34], we were able to obtain the optimized geometry of a sulfonamido-bridged dimeric complex represented in Figure 6. The structure is symmetric, with an inversion center in the middle of the distorted square formed by the two sulfonamido bridges. A detailed analysis of the metal ion environment shows a six-coordination pattern comprising the expected *N,N*,*O* binding site of a ligand unit, a sulfonamido *N*-bridge of the neighboring ligand unit, and two monodentate water molecules completing its coordination sphere. A comparison of this optimized structure with a related dimeric copper(II) complex, which is also bridged by sulfonamide N atoms, showed a great similarity. The reported complex shows Cu-N distances in the range 1.969(4)-2.004(4) Å, and bond angles in the range 85.12–98.4 [34].

Finally, with the aim of knowing the affinity of H_3_L to Cu^2+^ ions at room temperature, the value of the binding constant (K_B_) has been determined (Figure S9, K_B_ = intercept/slope). For this purpose, we used the Benesi–Hildebrand equation [36], using data from the UV-Vis absorption (K_B_ = 8.459 10^3^ M^−1^), as well as from the fluorescence emission intensity (K_B_ = 7.190 10^3^ M^−1^).

Since the reaction of H_3_L with Cu^2+^ ions resulted in a decrease of its fluorescence emission, we investigated the quenching mechanism with Stern-Volmer plots [37] (*F*_0_/*F* = 1 + *K*_SV_[Cu^2+^]). Figure S10 shows that the value of the quenching constant decreases with increasing temperature, which is a sign of static quenching. It must be noted that for static quenching (τ_0_/τ = 1), the *K*_SV_ value matches that of the K_B_ for the complex formation (*K*_SV_ = 9.010 10^3^ M^−1^). Hence, *K*_SV_ show similar values to K_B_ for the reaction of H_3_L with Cu^2+^ ions in a 1:1 molar ratio, supporting the accuracy of these measurements.

### 2.4. Investigations on the Interaction of H_3_L with CuO NPs

Spectrofluorimetry, Attenuated Total Reflection-Infrared spectroscopy (ATR-IR), Micro-X-ray Fluorescence (µ-XRF), Scanning Electron Microscopy-Energy Dispersive X-ray spectroscopy (SEM-EDX), and Density Functional Theory (DFT) were used to investigate the reaction product of H_3_L with CuO NPs.

#### 2.4.1. Spectrofluorimetry

**Figure 7:**
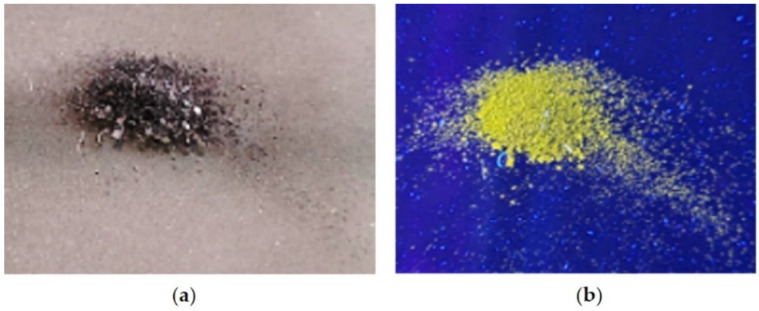
The effect of UV light (365 nm) on a powdery sample of H_3_L-CuO NPs; (**a**) under natural light, (**b**) under UV light (365 nm).

The interaction of H_3_L with CuO NPs leads to the emission of a yellow fluorescence when a solid sample of the reaction product is placed under a light source UV of 365 nm (Figure 7).

**Figure 8:**
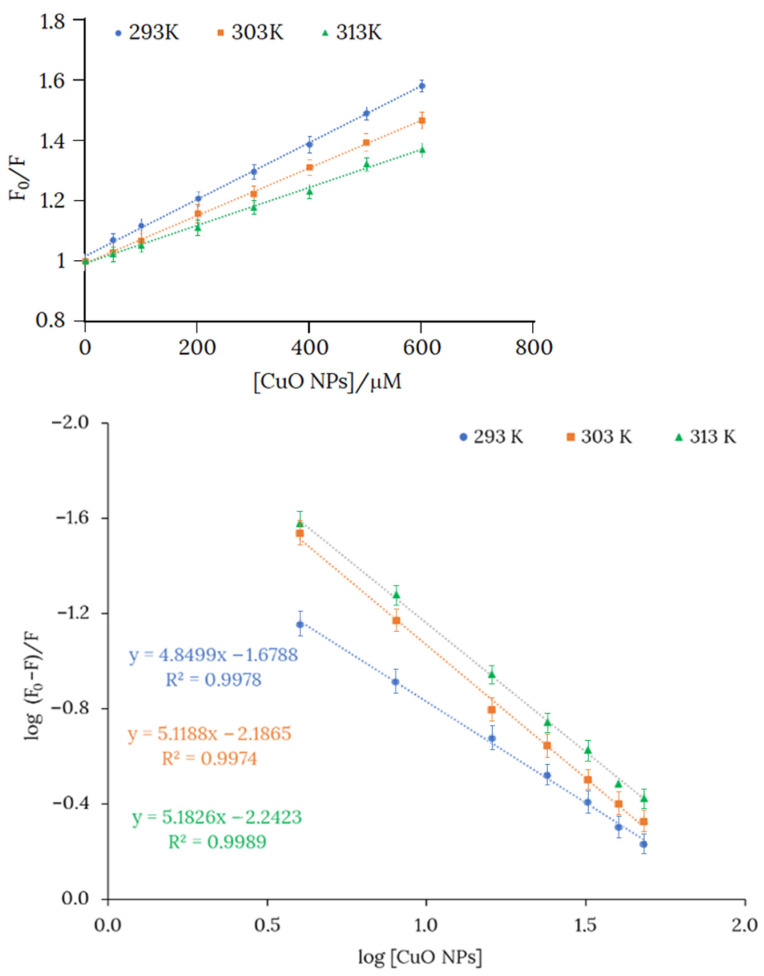
(**Top**): Plots of the intensities of the fluorescence spectra of H_3_L (10^−4^ M) vs. [CuO NPs] in an 80:20 ethanol:water solution. Slopes of the curves at 293, 303, and 313 K are Stern-Volmer constants *K*_SV_ (*K*_SV_ = *k*_q_τ, where τ is the lifetime of the fluorophore) at the cited temperatures. (**Bottom**): The Scatchard plot for binding H_3_L to CuO NPs at 293, 303, and 313 K and pH 11.

The fluorescence spectrum of the product obtained from the reaction between H_3_L and CuO NPs revealed a quenching of the fluorescence emission at 524 nm, in comparison with the fluorescence intensity of H_3_L measured under similar conditions. Stern-Volmer plots evidenced a static quenching mechanism, and therefore *K*_SV_ provides a measure of the bonding affinity between H_3_L and CuO NPs (Figure 8, top). As expected, *K*_SV_ for the reactions of H_3_L with CuO NPs and Cu^2+^ ions have similar values (Table 5). Values of Gibbs free energy, which have been determined from *K*_SV_, indicate that the dimeric copper(II) complex (−22.19 kJ mol^−1^) and H_3_L-CuO NPs (−22.41 kJ mol^−1^) also show similar stabilities.

The number of interaction binding sites (*n*) were determined by the Scatchard Equation (1), where *F*_0_ and *F* are the relative fluorescence of H_3_L in absence and presence of NMs, respectively, *K*_B_ is the binding constant, and [*Q*] is the quencher concentration [38]. The Scatchard equation linear graph of H_3_L and NMs is shown in Figure 8 (bottom). The number of binding sites (*n*) is around 5 at 293 K, suggesting five binding sites for H_3_L on the CuO NPs surface. The interaction ratio increased slightly with rising temperature, probably because temperature favors the reactivity of superficial Cu^2+^ ions.

(1) $$\log\left(\frac{F_{0}-F}{F}\right)=\log K_{B}+n\log\left[Q\right]$$

#### 2.4.2. Attenuated Total Reflection-Infrared Spectroscopy

**Figure 9:**
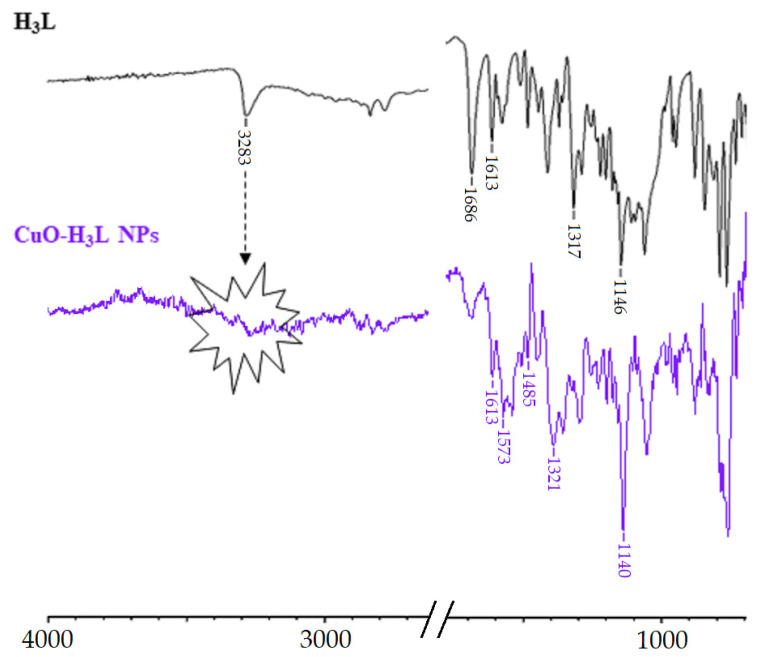
Partial view of the ATR-FTIR spectra of the free ligand (top) and CuO-H_3_L NPs.

The IR spectrum of H_3_L-CuO NPs clearly shows the absence of the band at about 3283 cm^−1^ attributable to ν OH/ν NH modes, which evidences the bideprotonation of the Schiff base ligand. Furthermore, a slight shift to lower wavenumbers of the bands corresponding to ν(NC), ν_as_(SO_2_), and ν_s_(SO_2_) can be observed The similarity between the ATR-IR spectra of CuO-H_3_L NPs and Cu_2_(HL)_2_(H_2_O)_4_·10H_2_O (Figure 9) indicates that the interaction between H_3_L and NMs surface occurs via metal-ligand coordination through the N_sulfonamide_, N_imine_, and O_phenol_ atoms. The observation of two bands attributable to ν_as_(COO^−^) and ν_s_(COO^−^) at about 1574 and 1391 cm^−1^, respectively, is a clear sign of the deprotonation of the carboxyl group present in the ligand, which is coherent with the formation of the carboxylate sodium salt.

#### 2.4.3. Micro-X-ray Fluorescence

**Figure 10:**
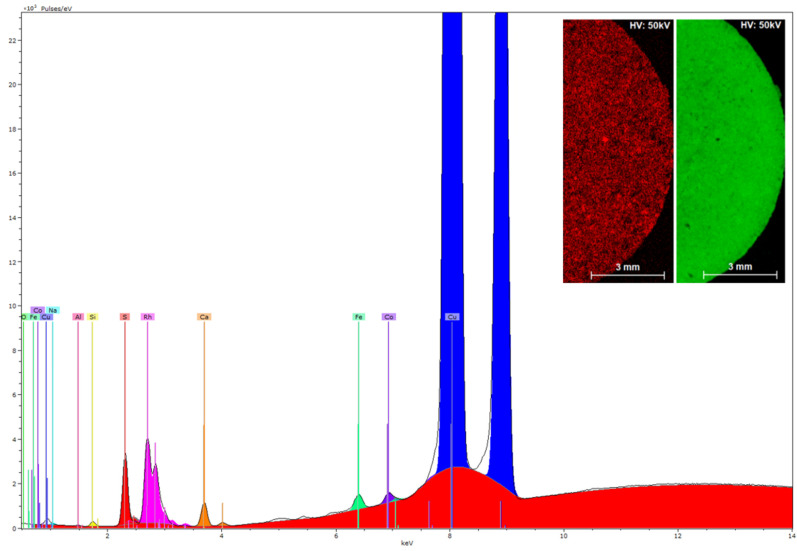
XRF spectrum of the reaction product of CuO NPs with H_3_L. *X* axis: energy (keV), *Y* axis: intensity. Inset: XRF mapping of the reaction product between CuO NPs and H_3_L with elemental distribution of sulfur (red) and copper (green) on the sample surface.

Figure 10 shows the peaks corresponding to the characteristic X-ray fluorescence emission of the chemical elements present in the reaction product of CuO NPs with H_3_L. The µ-XRF analysis indicates that the polycrystalline sample of CuO-H_3_L NPs contains copper and sulfur in a 30:1 ratio by weight, which is coherent with a 15:1 molar ratio (CuO:H_3_L). Moreover, µ-XRF mapping was used to explore the distribution of copper and sulfur within a sample of CuO-H_3_L NPs (Figure 10, inset). The homogeneity observed supports the effective interaction between the CuO NPs and H_3_L.

#### 2.4.4. Scanning Electron Microscopy-Energy Dispersive X-ray Spectroscopy

**Figure 11:**
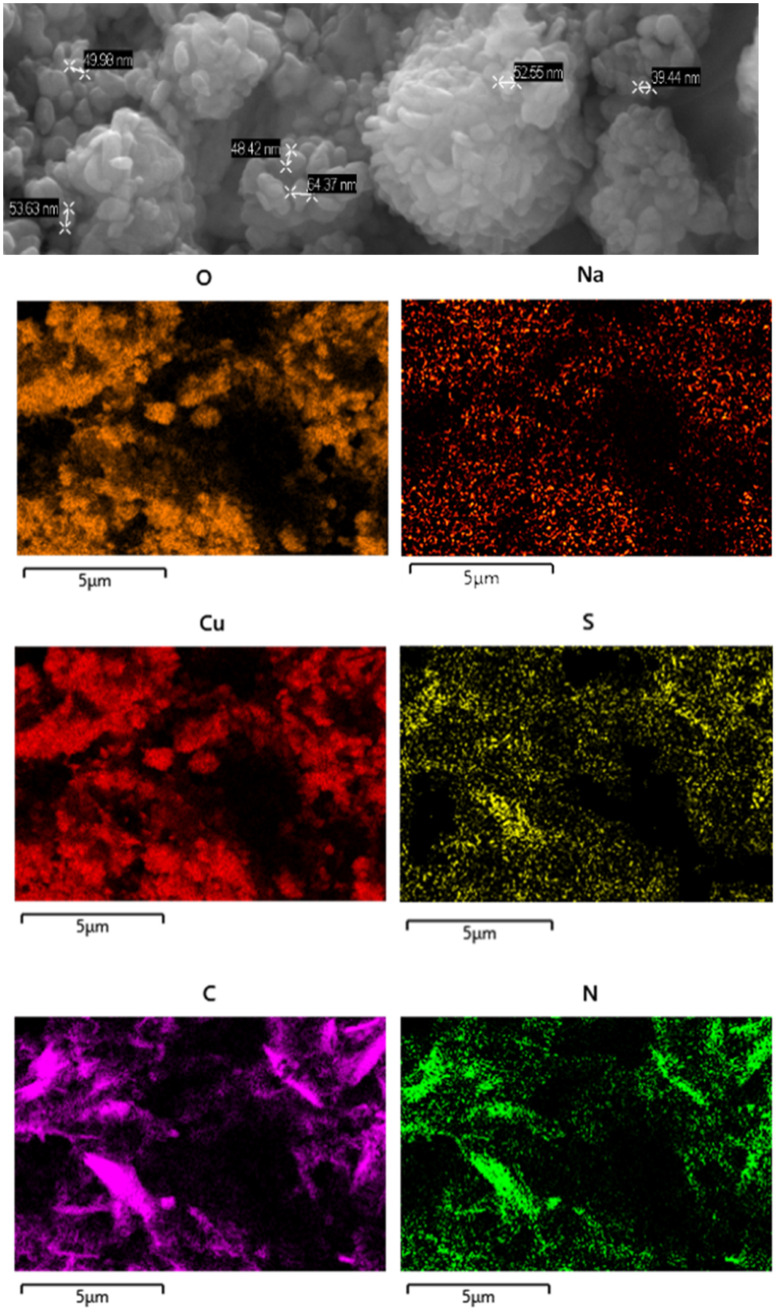
(**Top**): SEM micrograph of H_3_L-CuO NPs. (**Bottom**): EDX mapping pattern of the element distribution: Cu, O, S, N, C, and Na.

The SEM micrograph shown in Figure 11 show that the NPs are agglomerated to form clusters, which are made up of nanocrystals of sizes in the range of 70 to 40 nm, with a rather irregular shape.

A combination of SEM and EDX has been used to generate a “map” of element distributions in the raw sample. The EDX analysis shows the uniform presence of Cu, O, C, N, S, and Na elements with similar spatial distribution patterns, which supports the interaction of H_3_L (as carboxylate sodium salt) with CuO NPs.

#### 2.4.5. DFT Studies

**Figure 12:**
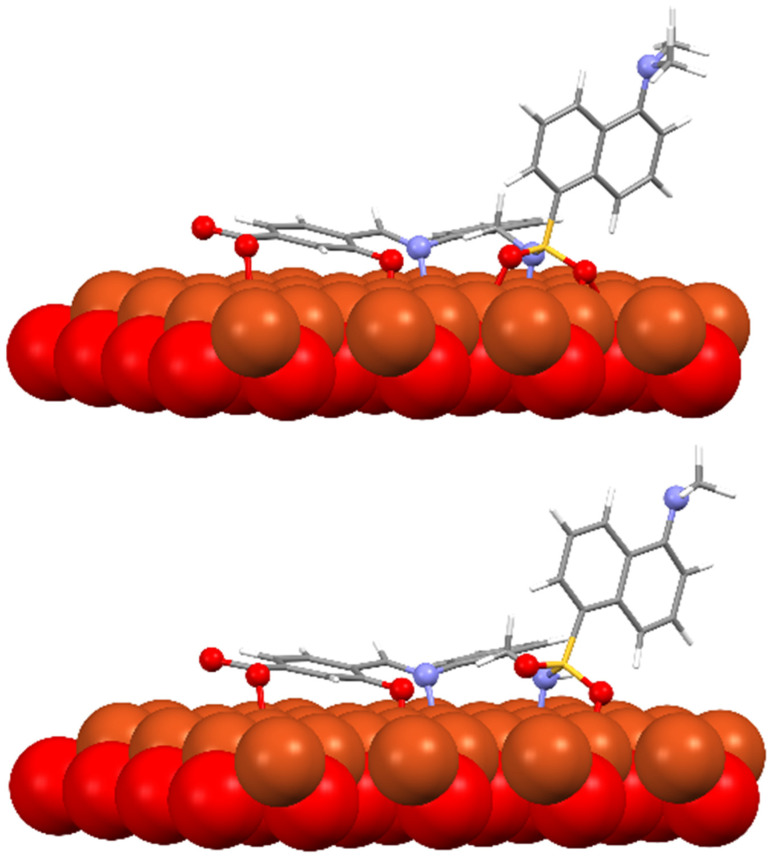
DFT models for the interaction H_3_L-NPs.

Several models have been considered to mimic the interaction between H_3_L and CuO NPs using DFT calculations [39] (Figure S11). The largest models studied comprise a single unit of the ligand interacting with a sheet of 32 units of CuO. The geometry of these surfaces has been taken from one of its common crystallographic structures (available from CSD) and choosing that facet containing the largest number of copper atoms suitable to interact with the ligand, in this case, the (1,0,0) facet. The size of the largest models has been chosen to allow a complete interaction between H_3_L and the NPs surface. Particular attention has been devoted to establishing the preference of a dianionic (as phenolate and sulfonamide anion) or a monoanionic (only as phenolate) form of the ligand to interact with the superficial Cu^2+^ ions on the NPs. Figure 12 shows the DFT models for the interaction of CuO NPs with these dianionic and monoanionic forms of the ligand.

As a result of these CuO NP models, we can deduce that the interactions of the dianionic form of the ligand show higher stabilization energies (−273.4 kcal/mol) than those corresponding to the monoanionic ones (−170.0 kcal/mol). A similar trend is observed for all the diverse models considered, even with the smallest models employed here, which only consider the interaction between the ligand and a single CuO unit (Figure S11). These theoretical results confirm that the interaction between H_3_L and the NMs surface takes place through the O_phenol_, N_imine_, and N_sulfonamide_ atoms of the ligand, as occurring for the dimeric copper(II) complex. In addition, the interactions of the superficial Cu^2+^ ions with the oxygen atoms of the carboxylate and the sulfonamide groups appear to contribute to the stabilization of the systems studied.

### 2.5. Fluorescence Emission Studies on H_3_L upon Addition of CuO NPs

**Figure 13:**
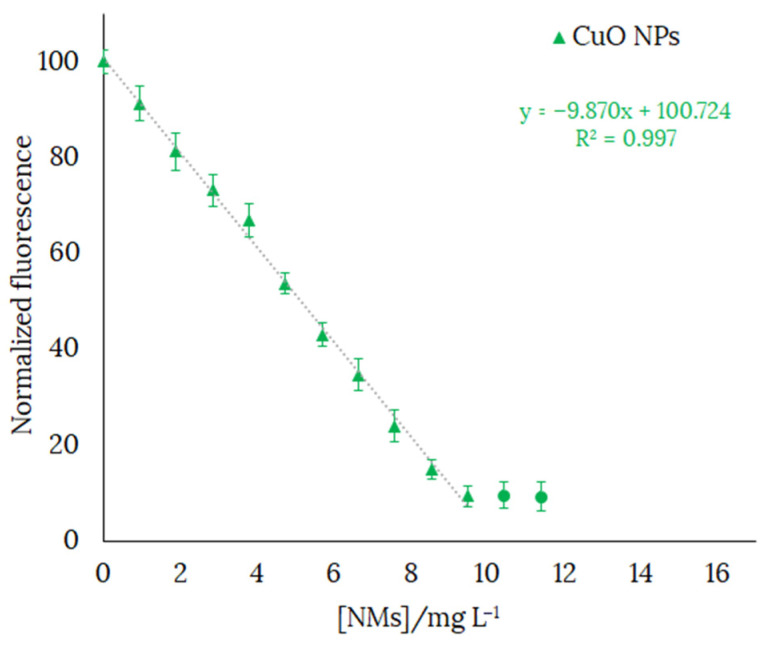
Fluorescence emission spectra calibration curve of H_3_L in 80:20 ethanol:water solution at pH 11, with gradual addition of CuO NPs (until the fluorescence intensity stops decreasing). [H_3_L] = 126 ppb. NaOH 0.1 M was used to deprotonate H_3_L. Final volume of the solution = 4 mL. Each solution was sonicated for 1 min before being measured. λ_exc_ = 400 nm.

In order to check the usefulness of H_3_L as a chemosensor for the detection of CuO NPs, we selected a concentration of H_3_L of 126 ppb. The fluorescence emission varied linearly in the range 0–10.5 ppm with the gradual addition of CuO NPs. Concentration values of CuO NPs higher than those indicated would oversaturate the system. The decrease by over 90% in the fluorescence emission of ethanol-water solutions (in 80:20 *v*/*v*) of H_3_L (λ_em_ = 540 nm) upon addition of CuO NPs (λ_em_ = 538 nm) is shown in Figure 13.

The limit of detection (LOD) and the limit of quantification (LOQ) of H_3_L have been expressed as LOD = 3SD/M and LOQ = 10SD/M, where SD is the standard deviation of the response and M is the slope of the calibration curve. LOD and LOQ were 330 ppb and 1.09 ppm, respectively. It must be noted that these values are higher than those previously reported by us for a pyrrole-based Schiff base ligand (13.83 μgL^−1^ and 46.05 μgL^−1^, respectively) [17] and a phenol-based Schiff ligand (9.8 μgL^−1^ and 32.6 μgL^−1^, respectively) [18]. The working range of H_3_L has been obtained using LOQ, as the minimum value that can be measured, and the highest value of [NMs] at which linearity is still maintained, as the maximum value. Therefore, the working range for detection of CuO NPs with [H_3_L] = 126 ppb was 1.1–9.5 ppm.

**Figure 14:**
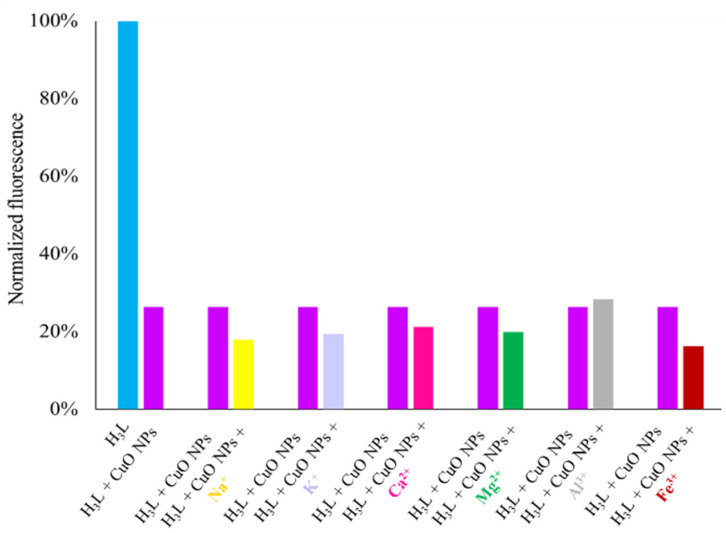
Fluorescence responses of H_3_L (2.5 nM, 1.26 ppb) toward CuO NPs (8 ppm) in the presence of some common metal ions (top) such as Na^+^ (8 ppm), K^+^ (8 ppm), Mg^2+^ (8 ppm), Ca^2+^ (8 ppm), Fe^3+^ (8 ppm), and Al^3+^ (4 ppm). All experiments were performed in 4 mL of an ethanol–water solution in 80:20 *v/v* (pH 11) under λ_exc_ = 400 nm.

In view of the affinity of H_3_L to CuO NPs, we studied the selectivity of H_3_L as a probe for the cited NPs. As a criterion for interference, a ±10% variation of the average fluorescence intensity was used. The selectivity of H_3_L as a probe for CuO NPs was tested in the presence of common metal ions in water, such as Na^+^, K^+^, Mg^2+^, Ca^2+^, Fe^3+^, and Al^3+^ metal ions, in the same concentration as CuO NPs (Figure 14). These results showed that the cited species do not significantly interfere with the detection of CuO NPs. Al^3+^ ions can be tolerated in concentrations of at least 4 ppm without interference.

## 3. Materials and Methods

All starting materials and reagents were commercially available, and they were used without further purification. ^1^H NMR spectra (400 MHz) were measured in deuterated solvents using a Varian Inova 400 Spectrometer. *J* values are given in Hertz. The IR spectra were measured in the range 4000 to 400 cm^−1^ wavenumber using a FTIR spectrometer PerkinElmer Spectrum Two coupled with Platinum Diamond ATR, which consists of a diamond disc as an internal reflection element. Elemental analyses were performed on an elemental analyzer FISONS model EA 1108. Fluorescence emission studies were performed on a Shimadzu RF-600 Spectro Fluorophotometer. X-ray fluorescence was measured under vacuum (19.6 mbar) using an M4 TORNADO system (BRUKER), with the Rh tube, operating at 50 kV and 200 µA. The fluorescence maps were collected for a total time of 2 ms per pixel, with a pixel size of 20 µm. Scanning Electron Microscopy (SEM) was applied to investigate the size and morphology of the NPs, and it was carried out under a ZEISS FESEM ULTRA Plus with EDX microanalysis.

### 3.1. Sample Preparation

Samples for investigations on the interaction of H_3_L with CuO NPs were obtained by stirring an ethanol solution of H_3_L at pH 9, and a suspension of NPs in a 2:1 molar ratio, at room temperature for about 15 min. Then, the sample was air-dried for about 48 h.

### 3.2. Crystal Structure Analysis Data

Diffraction data for prismatic crystals corresponding to DsA and H_3_L were collected at 100(2) K, using graphite-monochromatized Mo-*Kα* radiation (λ = 0.71073 Å) from a fine focus sealed tube, using a Bruker D8 Venture Photon III-14 diffractometer. Data were routinely processed and corrected for Lorentz and polarization effects. Multi-scan absorption corrections were performed using the SADABS routine [40]. The structure was solved by standard direct methods using SHELXT [41], and then refined by full matrix least squares on *F*^2^ by using the SHELXL program [42]. All non-hydrogen atoms were anisotropically refined. Hydrogen atoms were mostly included in the structure factor calculation in geometrically idealized positions, with thermal parameters depending on the parent atom, by using a riding model. H atoms potentially involved in H-bonding were localized in Fourier maps and then refined with a thermal parameter depending on the parent N atom. More details of the refinement, as well as crystal data are collected in Table S1 of the ESI.

CCDC 2,121,699 and 2,121,700 contain the supplementary crystallographic data for this paper. These data can be obtained free of charge from the Cambridge Crystallographic Data Centre via www.ccdc.cam.ac.uk/data_request/cif (accessed on 20 September 2022)

### 3.3. Computational Methods

Theoretical calculations on DsA, H_3_L, copper(II) complex, and ligand-NPs models have been performed employing density functional theory (DFT) methods, as implemented in Gaussian 09 [39]. Thus, M062X/6-31G* geometrical optimization of different starting structures of DsA, H_3_L, and its copper(II) complex have been performed. The most stable conformers found have been characterized by calculation of their vibrational frequencies. For DsA and H_3_L, we tested the idea that the M062X/6-31G* level provided results of comparable quality to those obtained with a more extended basis set as 6-311++G** (not included for brevity), but with a significantly smaller computational cost. For comparison with the experiment results, the theoretical IR spectra of DsA and H_3_L have been obtained using the harmonic vibrational frequencies scaled by 0.947 and a FWHM of 4 cm^−1^.

The electronic spectra of DsA and H_3_L have been obtained employing the M062X/6-31G* level, and the influence of the solvent has been included using the PCM method and ε = 24.852 for ethanol. The main electronic transitions have been analyzed by computing their natural transition orbitals, which provide a picture of the orbitals involved in any electronic transition, which is easier to interpret [43].

Models of different size have been used to evaluate the interaction of the ligand with CuO NPs. The geometry for the smallest model, comprising only one H_3_L unit and a single CuO unit, has been optimized without restrictions. In the remaining cases, the geometries of the ligand over the surface have been freely optimized, keeping a frozen structure for the metal obtained from crystallographic data.

Interaction energies, E_int_, for these systems have been evaluated as E_int_ = E_complex_ − (E_H__3L_ + E_metal model_), where the E_complex_ is the total energy of the interacting ligand-NPs, E_H__3L_ is the total energy of the monoanionic or dianionic form of the H_3_L ligand, and E_metal_ is the energy of the different metal models indicated above. These interaction energies have been evaluated by means of the NEDA method included in the NBO7.0 program [44], so that the basis set superposition error is corrected by means of the counterpoise method.

### 3.4. Synthesis and Characterization of Compounds

#### 3.4.1. DsA

The compound *N*-(2-aminobenzyl)-5-(dimethylamino)naphthalene-1-sulfonamide was prepared following a synthetic procedure previously used with imidazolopyridine compounds [45], but with some modifications. In a 250 mL round flask, 2.35 mmol (0.6339 g) of dansyl chloride was dissolved in 60 mL of CH_2_Cl_2_. In a second flask, 2.59 mmol (0.3162 g) of the 2-aminobenzylamine was dissolved in 40mL of CH_2_Cl_2_, at which 2.59 mmol (0.2631 g) of triethylamine was added. The content of the second flask was added to the dansyl chloride solution, giving a dark yellow solution which was stirred at room temperature for 18 h. During this time period, the solution changed its color from dark to light yellow. Evaporation under reduced pression of the solution to a third of its volume, and the addition of diethyl ether until the observation of turbidity, led to the formation of a light brown precipitate, which is a small unreacted 2-aminobenzylamine fraction. Filtration of the mixture led to the separation of 2-aminobenzylamine as a powdery solid. Evaporation to dryness under vacuum of the filtrate led to the precipitation of a light yellow powdery solid, which was washed with deionized water to separate triethylamine hydrochloride. The yellow solid was filtrated and air-dried to be characterized as DsA.

Yield: 0.793 g (95%). ^1^H NMR (400 MHz, dmso-*d_6_,* δ in ppm): δ 8.47 (d, 1H), 8.35 (d, 1H), 8.24 (t, 1H), 8.14 (d, 1H), 7.62 (t, 1H), 7.60 (t, 1H), 7.27 (d, 1H), 6.94 (d, 1H), 6.92 (t, 1H), 6.58 (d, 1H), 6.43 (t, 1H), 4.87 (s, 2H), 3.85 (d, 2H), 2.84 (s, 6H). UV-Vis (ethanol, 4.02 10^−5^ mol L^−1^, *λ* in nm, *ε* in parenthesis) 252 (67,262 M^−1^cm^−1^), 294 (14,754 M^−1^cm^−1^), 346 (19,322 M^−1^cm^−1^). Fluorescence λ/nm: λ_em_ 520 (λ_ex_ 336, ethanol, bandwidth of 5 nm). ATR-FTIR (ν in cm^−1^): 3472 w (ν_as_ HNH), 3384 m (ν_s_ HNH), 3323 m (ν NH), 1632 m (δ HNH), 1309 s (ν_as_ OSO), 1143 s (ν_s_ OSO). Elemental analysis (found): C 63.8; H 5.9; N 11.8; S 8.5%; Calc. for C_19_H_21_N_3_O_2_S: C, 64.2; H, 6.0; N, 11.8; S, 9.0% (Mw: 355.5 g·mol^−1^).

#### 3.4.2. H_3_L

H_3_L was prepared following a synthetic procedure previously used by us [19]. In a 250 mL round-bottomed flask, 0.104 g of DsA (0.29 mmol) was dissolved in 60 mL of absolute ethanol. Then, while the solution was being stirred, 4-formyl-3-hydroxybenzoic acid (0.034 g, 0.29 mmol) in absolute ethanol (30 mL) was added. A light-yellow solution was obtained, which was stirred at room temperature for 24 h. Then, the solution was concentrated in a rotary evaporator to dryness, and a yellow precipitate was obtained. Then, hexane was added to wash the precipitate. This was filtered under vacuum and an orangey-colored precipitate is obtained, which was left to dry under vacuum for a couple of hours. The crude solid was purified by column chromatography. We used hexane:ethyl acetate (50:50) to extract the unreacted DsA, and methanol to extract H_3_L.

Yield: 0.120 g (82%); ^1^H NMR (400 MHz, dmso-*d*_6_) δ 13.18 (s, 1H), 12.29 (s, 1H), 8.66 (s, 1H), 8.38 (t, 1H), 8.36 (d, 1H), 8.26 (d, 2H), 8.02 (d, 1H), 7.72 (d, 1H), 7.49 (d, 1H), 7.48 (t, 1H), 7.47 (t, 1H), 7.44 (s, 1H), 7.27 (d, 1H), 7.23 (t, 1H), 7.19 (d,1H), 7.09 (d, 1H), 7.08 (t, 1H), 4.19 (d, 2H), 2.81 (s, 6H). UV-Vis (absolute ethanol, 10^−3^ mol L^−1^, *λ* in nm, *ε* in parenthesis) 224 (2,770 M^−1^cm^−1^), 248 (1,743 M^−1^cm^−1^), 260 (1,550 M^−1^cm^−1^), 340 (711 M^−1^cm^−1^). Fluorescence λ/nm: λ_em_ = 510 (λ_ex_ = 400, ethanol, bandwidth of 5 nm). ATR-IR (ν in cm^−1^): 3283 m (ν NH), 1686 s (ν CO), 1613 m (ν CN), 1412 m (δ COH), 1317 s (ν_as_ OSO), 1146 s (ν_s_ OSO). Elemental analysis (found): C 57.0; H 4.8; N 7.3; S 5.3%; Calc. for C_27_H_25_N_3_O_5_S·HCl·H_2_O: C, 58.0; H, 5.4; N, 7.5; S, 5.7% (Mw: 558.1 g·mol^−1^).

#### 3.4.3. Cu_2_(HL)_2_(H_2_O)_4_·10H_2_O

Method 1: The copper(II) complex was obtained at room temperature by electrochemical oxidation of a metal anode immersed in an acetonitrile solution (75 mL) of H_3_L (0.05 g, 0.01 mmol), containing tetraethylammonium perchlorate (ca. 20 mg) as a supporting electrolyte (Caution: Although no problem has been encountered in this work, all perchlorate compounds are potentially explosive and should be handled in small quantities and with great care!). This was electrolyzed for about 1h and 3 min at a current intensity of 5.0 mA and an initial voltage of 9.4 V. The suspension so obtained was concentrated in a rotary evaporator to dryness, and a dark-green precipitate was obtained. Then, the powdery solid was washed with deionized water, filtered, and air-dried (yield = 54%).

Method 2: An ethanol solution (60 mL) of H_3_L (0.04 g, 0.08 mmol) was added to 50 mL of an ethanol solution of Cu(OAc)_2_·H_2_O (0.0158 g, 0.0794 mmol). The resulting solution was stirred in a round-bottomed flask of 250 mL for 3 h at room temperature. The color of the solution changed from pale yellow to dark brown during the reaction time. The suspension obtained was evaporated to dryness under vacuum. Then, the powdery solid was washed with diethyl ether, filtered, and air-dried (yield = 37%).

ATR-IR (ν in cm^−1^): 3393 br (ν OH, ν NH), 1612 m (ν CN), 1574 s (ν_as_ OCO), 1392 s (ν_s_ OCO), 1314 s (ν_as_ OSO), 1119 s (ν_s_ OSO). UV-Vis (ethanol, *λ* in nm) 219, 244, 336, 414. Fluorescence λ/nm: λ_em_= 530 (λ_ex_= 400, ethanol, bandwidth of 15 nm). Elemental analysis (found): C 46.9; H 5.3; N 6.3; S 4.6%; calc. for C_54_H_54_Cu_2_N_6_O_14_S_2_·10H_2_O: C, 46.9; H, 5.4; N, 6.1; S, 4.6 (Mw: 1382.4 g mol^−1^). 

## 4. Conclusions

The crystal structures of H_3_L and DsA were elucidated, and a detailed assignment of the most significant bands of both UV-Vis and infrared spectra has been done on the basis of the calculated spectra using density functional theory (DFT) methods. The DFT-optimized geometry of Cu_2_L_2_(H_2_O)_4_ shows a six-coordination environment comprising a *N*,*N*,*O* binding site of a ligand unit, a sulfonamido N-bridge of the neighboring ligand unit, and two water molecules completing its coordination sphere. The affinity of H_3_L to react with Cu^2+^ ions in solution, giving rise to Cu_2_L_2_(H_2_O)_4_ (K_B_ = 9.01 10^3^ L mol^−1^), is similar to that found for the interaction with Cu^2+^ ions on the surface of CuO NPs (K_B_ = 9.84 10^3^ L mol^−1^). Values of Gibbs free energy indicate that the dimeric copper(II) complex (−22.19 kJ mol^−1^) and H_3_L-CuO NPs (−22.41 kJ mol^−1^) also have similar stabilities. Fluorescence spectroscopic measurements suggest five binding sites for H_3_L on the CuO NPs surface. The µ-XRF analysis indicates that the polycrystalline sample of CuO-H_3_L NPs contains copper and sulfur in a 30:1 ratio by weight, which is coherent with a 15:1 molar ratio (CuO:H_3_L). The EDX mapping of the element distribution shows the uniform presence of Cu, Na O, C, N, and S elements with a similar spatial distribution pattern, supporting the existence of the interaction of H_3_L (as carboxylate sodium salt) with CuO NPs. ATR spectroscopy shows the bideprotonation of the Schiff base ligand, as well as a slight shift to lower wavenumbers of the bands corresponding to ν(NC), ν_as_(SO_2_), and ν_s_(SO_2_), which demonstrates the chelating behavior of the Schiff base ligand. DFT studies confirm that the interaction between H_3_L and the NMs surface takes place through the O_phenol_, N_imine_, and N_sulfonamide_ atoms of the dianionic ligand (as phenolate and sulfonamide anion), as occurring for the copper(II) complex.

We probed that at room temperature and basic pH, the fluorescence intensity of H_3_L in aqueous solution decreased linearly with increasing concentration of CuO NPs. A solution of H_3_L (126 ppb) shows sensitive responses to CuO NPs, with a limit of detection (LOD) as low as 330 ppb, and a working range between 1.1 and 9.5 ppm. H_3_L possesses selectivity toward CuO NPs in the presence of common metal ions in water, such as Na^+^, K^+^, Mg^2+^, Ca^2+^, Fe^3+^, and Al^3+^ ions.

## Figures and Tables

**Table 1:** Molecular orbitals involved in the main transitions originating the UV-Vis spectrum of DsA.

Wavelength (nm)	Oscillator Strength	Molecular Orbitals		Natural Transition Orbitals ^a^		
296.2	(0.1719)	HOMO-1 → LUMO	95.9%	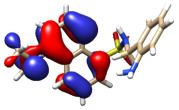	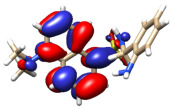	(96%)
220.8	(0.3030)	HOMO-1 → LUMO+1 HOMO-4 → LUMO HOMO-3 → LUMO+1 HOMO-3 → LUMO	38.3% 31.9% 13.1% 7.5%	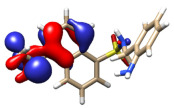	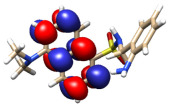	(56%)
				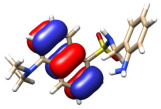	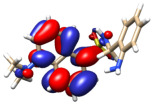	(39%)
195.8	(0.3430)	HOMO-3 → LUMO+1 HOMO-6 → LUMO HOMO-4 → LUMO	46.3% 25.2% 9.3%	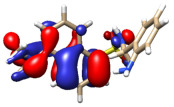	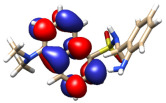	(50%)
				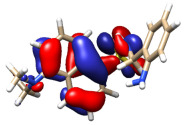	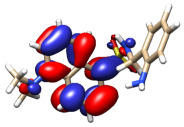	(37%)
187.1	(0.4146)	HOMO-4 → LUMO+1 HOMO-3 → LUMO+1 HOMO-1 → LUMO+3	73.4% 8.8% 6.2%	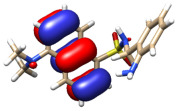	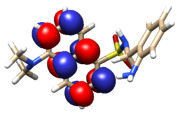	(83%)
174.6	(0.2974)	HOMO-2 →LUMO+2 HOMO → LUMO+4 HOMO → LUMO+5 HOMO-8 → LUMO	55.4% 9.0% 7.7% 7.1%	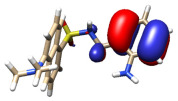	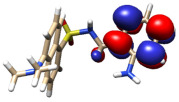	(55%)
				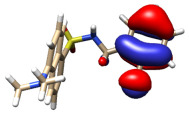	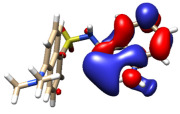	(18%)

**^a^** The contributions of the represented NTO pairs to the electronic transitions are given between parenthesis.

**Table 2:** Molecular orbitals involved in the three more intense transitions originating the UV-vis spectrum of H_3_L.

Wavelenght (nm)	Oscillator Strength	Molecular Orbitals		Natural Transition Orbitals ^a^		
323.4	(0.42700)	HOMO-1 → LUMO HOMO → LUMO	72.3% 19.6%	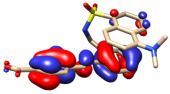	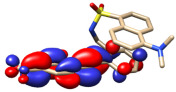	(92%) ^a^
263.3	(0.26370)	HOMO-6 → LUMO HOMO-2 → LUMO HOMO-10 → LUMO HOMO-1 → LUMO HOMO-9 → LUMO	26.7% 25.4% 12.3% 6.4% 6.1%	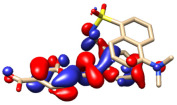	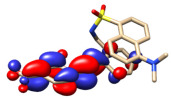	(84%)
223.7	(0.18510)	HOMO-4 → LUMO+1 HOMO → LUMO+2 HOMO-4 → LUMO HOMO-3 → LUMO+1 HOMO → LUMO+4	27.0% 25.1% 6.5% 5.7% 5.4%	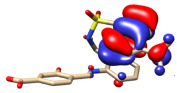	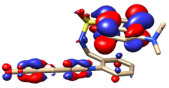	(42%)
				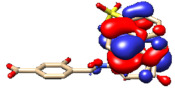	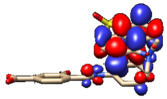	(33%)

^a^ The contributions of the represented NTO pairs to the electronic transitions are given between parenthesis.

**Table 3:** Experimental and theoretical vibrational frequencies (cm^−1^) of H_3_L.

Experimental ^a^	Theoretical ^a^	Normal Vibrational Mode Assignment ^a^
1145	1109	O=S=O symm stretch
1317	1303	O=S=O asymm stretch + N-H bending + CH_2_ bending
1412	1377	C-O-H bending (phenolic ring)
1613	1642	N=C stretch
1686	1796	C=O stretch (COOH group)
3283	3204	O-H stretch (phenol)

^a^ The unsigned mean error for these assignments is 43 cm^−1^.

**Table 4:** Experimental and theoretical vibrational frequencies (cm^−1^) of DsA.

Experimental ^a^	Theoretical ^a^	Normal Vibrational Mode Assignment ^a^
1142	1113	O=S=O symm stretch + ring C-H bending (aniline + dansyl)
1310	1310	O=S=O asymm stretch + N-H bending (aniline) + CH_2_ twist (aniline)
1630	1601, 1624	NH_2_ bending (aniline) + C=C asymm stretch (aniline)
3323	3367	N-H stretch (aniline)
3383	3389	NH_2_ symm stretch (aniline)
3470	3484	NH_2_ asymm stretch (aniline)

^a^ The unsigned mean error for these assignments is 21 cm^−1^.

**Table 5:** Values of Gibbs free energy, binding constant, and quenching constant for the reaction of H_3_L with Cu^2+^ ions (in 1:1 molar ratio), as well as CuO NPs. Binding constants were obtained from fluorescence emission intensity and UV-Vis absorption.

Species	ΔG *_K_*_sv_ kJ mol^−1^	K_b FL_ M^−1^	K_b UV-Vis_ M^−1^	*K*_sv 293 K_ M^−1^	*K*_sv 303 K_ M^−1^	*K*_sv 313 K_ M^−1^
Cu^2+^	−22.19	7190	8459	9010	7650	6420
CuO NPs	−22.41	-	-	9840	8470	6920

## Data Availability

Not applicable.

## Supplementary Materials

The following supporting information can be downloaded at: https://www.mdpi.com/article/10.3390/ijms231911565/s1.
