# Supplementary material for: Experimental and Computational Studies on the Interaction of a Dansyl-Based Fluorescent Schiff Base Ligand with Cu2+ Ions and CuO NPs
Source: Int J Mol Sci. 2022 Sep 30;23(19):11565. doi: 10.3390/ijms231911565 (PMC9569476; doi:10.3390/ijms231911565)
Supplement: Supplementary file 1 [file ijms-23-11565-s001.zip › ijms-1911388-supplementary.pdf]

Supplementary Materials for:

Experimental and computational studies on the interaction of a dansyl–based fluorescent Schiff base ligand with Cu^{2+} ions and CuO NPs.

Jesús Sanmartín-Matalobos [a], Pilar Bermejo-Barrera [b], Ignacio Pérez-Juste,[c] Matilde Fondo [a] Ana M. García-Deibe *[a] and Yeneva Alves-Iglesias [a,b]

*ana.garcia.deibe@usc.es

Table of Contents.. page

1. NMR Spectra of DsA and H_3L..........S2
2. Crystal and refinement data for DsA and $[H_4L]Cl·0.92H_2O$..........S3
3. Selected bond lengths [Å] and angles [°] for DsA and $[H_4L]Cl·0.92H_2O$..........S4
4. Torsion angles [°] for DsA and $[H_4L]Cl·0.92H_2O$..........S4
5. Hydrogen bonds for $[H_4L]Cl·0.92H_2O$ [Å and deg.]..........S5
6. Optimized structure for the H_3L dimer..........S6
7. Bond length and bond angles differences between experimental and theoretical values for H_3L..........S7
8. Hydrogen bonds for DsA [Å and deg.]..........S8
9. Optimized structure for DsA..........S9
10. Bond length and bond angles differences between experimental and theoretical values for DsA..........S10
11. Infrared study of H_3L..........S11-S12
12. Infrared study on DsA..........S13-S14
13. ATR-FTIR spectrum of the copper(II) complex..........S15
14. UV-Vis spectrum of the copper(II) complex..........S16
15. Benesi–Hildebrand plot of H_3L with Cu^{2+}..........S17
16. Stern-Volmer plot of H_3L *vs* $[Cu^{2+}]$..........S18
17. DFT study of interaction of H_3L with of CuO NPs..........S19

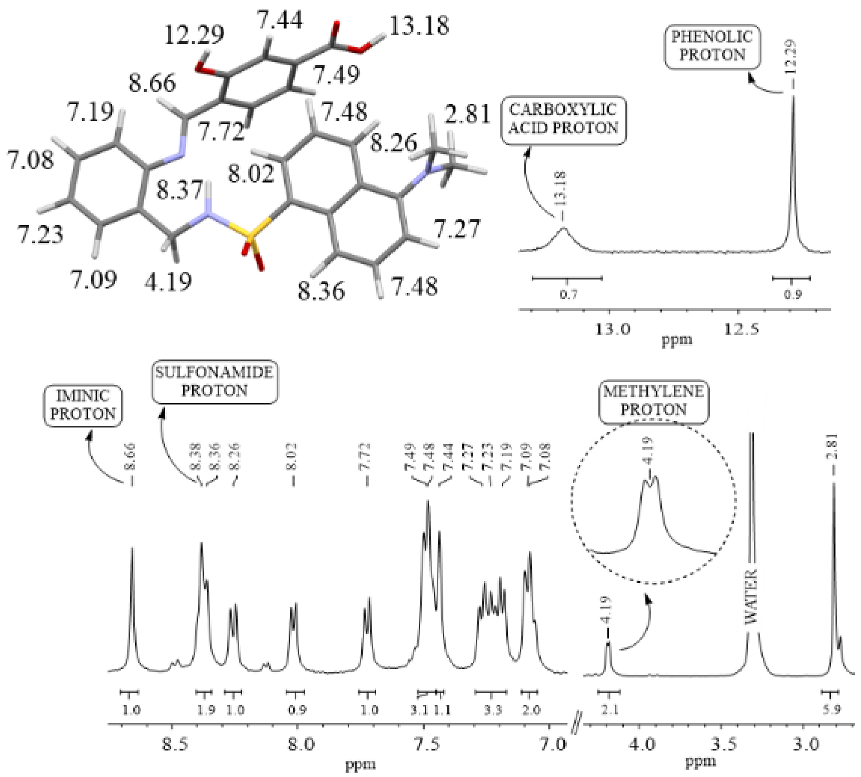

Figure S1. View of the ^1H NMR spectrum of H_3L (in dmso-d_6), with signal assignment on a 3D model of the molecule.

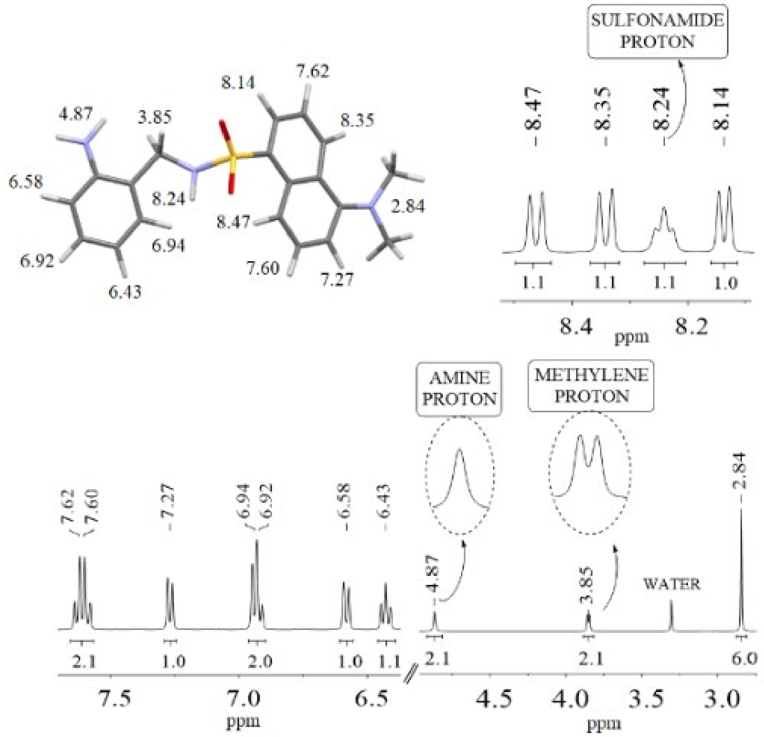

Figure S2. ^1H NMR spectrum of DsA (in dmso-d_6), with signal assignment on a 3D model of the molecule.

Table S1. Crystal and structure refinement data for DsA and $[H_4L]Cl·0.92H_2O$

	DsA	$[H_4L]Cl·0.92H_2O$
Empirical formula	$C_{19}H_{21}N_3O_2S$	$C_{27}H_{27.83}ClN_3O_{5.92}S$ 556.52
Formula weight	355.45	100(2) K
Temperature	100(2) K	0.71073 Å
Wavelength	1.54178 Å Monoclinic	Triclinic
Crystal system	*C*2/*c*	*P*-1
Space group	*a* = 16.6384(3) Å	*a* = 9.8245(3) Å
Unit cell dimensions	*b* = 9.5135(2) Å	*b* = 16.0619(5) Å
	c = 22.8392(5) Å	*c* = 17.8841(6) Å
	α = 90 °	α = 101.4218(15)°
	β = 108.1050(10) °	β = 102.8605(16)°
	γ = 90 °	γ = 104.0848(15)°
	3436.21(12) Å^3	2571.94(14) Å^3
Volume	8	4
Z	1.374 Mg/m^3	1.437 Mg/m^3
Density (calculated)	1.820 mm^{-1}	0.278 mm^{-1}
Absorption coefficient	1504	1165
F(000)	0.09 x 0.09 x 0.04	0.165 x 0.112 x 0.026 mm^3
Crystal size	mm^3 4.073 to 74.486 °	2.214 to 28.333 °.
Theta range for data collection		
Index ranges	-20 ≤ *h* ≤ 20,	-13 ≤ *h* ≤ 13,
	-11 ≤ k ≤ 11,	-21 ≤ k ≤ 21,
	-28 ≤ *l* ≤ 28	-23 ≤ *l* ≤ 23
Reflections collected	43634	106750
Independent reflections	3504 (R_{int} = 0.0600)	12816 (R_{int} = 0.0677)
Completeness to theta = 25.242°	100 %	99.9 %
Refinement method	Full-matrix least-squares on F^2	Full-matrix least-squares on F^2
Data / restraints / parameters	3504 / 0 / 240	12816 / 499 / 891
Goodness-of-fit on F^2	1.099	1.095
Final R indices [I>2sigma(I)]	R_1 = 0.0430, wR_2 = 0.0932	R_1 = 0.0590, wR_2 = 0.1320
R indices (all data)	R_1 = 0.0484, wR_2 = 0.0959	R_1 = 0.0741, wR_2 = 0.1392
Largest diff. peak and hole	0.396 and -0.3637 e.Å^{-3}	0.733 and -0.754 e.Å^{-3}

Table S2. Selected bond lengths [Å] and angles [°] for DsA and $[H_4L]Cl·0.92H_2O$

**DsA**		**$H_3L·0.92H_2O$**			
		O(1)-C(1)	1.341(3)	O(41)-C(41)	1.342(3)
		C(7)-O(8)	1.196(3)	C(47)-O(48)	1.215(3)
		C(7)-O(9)	1.314(3)	C(47)-O(49)	1.327(3)
		C(10)-N(11)	1.282(3)	C(50)-N(51)	1.286(3)
N(11)-C(12)	1.379(3)	N(11)-C(12)	1.420(3)	N(51)-C(52)	1.423(3)
C(18)-N(19)	1.459(2)	C(18)-N(19)	1.479(3)	C(58)-N(59)	1.484(3)
N(19)-S(20)	1.6135(17)	N(19)-S(20)	1.6116(18)	N(59)-S(60)	1.6080(19)
S(20)-O(22)	1.4318(14)	S(20)-O(22)	1.4375(16)	S(60)-O(61)	1.4339(16)
S(20)-O(21)	1.4427(14)	S(20)-O(21)	1.4385(16)	S(60)-O(62)	1.4388(16)
S(20)-C(23)	1.7775(19)	S(20)-C(23)	1.773(2)	S(60)-C(63)	1.791(5)
C(28)-N(33)	1.420(2)	C(28)-N(33)	1.483(3)	C(68)-N(73)	1.485(4)
N(33)-C(35)	1.463(2)	N(33)-C(35)	1.493(3)	N(73)-C(74)	1.491(4)
N(33)-C(34)	1.453(2)	N(33)-C(34)	1.497(3)	N(73)-C(75)	1.505(4)
		O(1)-C(1)-C(2)	122.2(2)	O(41)-C(41)-C(42)	122.6(2)
		O(8)-C(7)-O(9)	123.6(2)	O(48)-C(47)-O(49)	123.1(2)
		C(10)-N(11)-C(12)	123.46(19)	C(50)-N(51)-C(52)	121.9(2)
N(19)-C(18)-C(17)	113.01(16)	N(19)-C(18)-C(17)	111.27(16)	N(59)-C(58)-C(57)	112.60(17)
C(18)-N(19)-S(20)	120.30(14)	C(18)-N(19)-S(20)	121.09(15)	C(58)-N(59)-S(60)	120.65(16)
O(22)-S(20)-O(21)	118.47(8)	O(22)-S(20)-O(21)	117.74(10)	O(61)-S(60)-O(62)	117.94(10)
N(19)-S(20)-C(23)	108.22(9)	N(19)-S(20)-C(23)	106.47(10)	N(59)-S(60)-C(63)	107.4(3)
C(28)-N(33)-C(35)	114.86(15)	C(28)-N(33)-C(35)	112.9(2)	C(68)-N(73)-C(74)	114.8(3)
C(28)-N(33)-C(34)	115.10(16)	C(28)-N(33)-C(34)	113.8(2)	C(68)-N(73)-C(75)	110.6(3)
C(35)-N(33)-C(34)	110.49(16)	C(35)-N(33)-C(34)	110.5(2)	C(74)-N(73)-C(75)	110.5(3)

Table S3. Torsion angles [°] for for DsA and $[H_4L]Cl·0.92H_2O$

**DsA**		**$H_3L·0.92H_2O$**	
C(18)-N(19)-S(20)-C(23)	64.68(16)	C(18)-N(19)-S(20)-C(23)	-47.85(18)
		C(58)-N(59)-S(60)-C(63)	52.0(2)

Table S4. Hydrogen bonds for $[H_4L]Cl\cdot0.92H_2O$ [Å and °].

D-H···A	d(D-H)	d(H···A)	d(D···A)	<(DHA)
O(1)-H(1)...N(11)	0.82(3)	1.83(3)	2.588(3)	153(3)
O(9)-H(9)...Cl(2^a)	0.92(3)	2.14(4)	3.051(2)	169(3)
O(9)-H(9)...Cl(2B^b)	0.92(3)	2.06(4)	2.923(14)	155(3)
O(9)-H(9)...Cl(2C^c)	0.92(3)	2.16(4)	2.975(17)	147(3)
O(9)-H(9)...Cl(2D^d)	0.92(3)	2.20(4)	3.10(3)	165(3)
N(19)-H(19)...N(11)	0.82(3)	2.61(3)	3.000(3)	111(2)
N(19)-H(19)...O(62)	0.82(3)	2.18(3)	2.975(2)	164(3)
N(33)-H(33)...Cl(1)	0.96(3)	2.09(3)	3.014(2)	160(3)
O(41)-H(41)...N(51)	0.84(3)	1.90(3)	2.650(3)	148(3)
O(49)-H(49)...Cl(1)#1	0.89(3)	2.14(3)	3.018(2)	172(3)
N(59)-H(59)...O(21)	0.79(3)	2.22(3)	2.983(2)	162(3)
N(59)-H(59)...N(51)	0.79(3)	2.55(3)	2.997(3)	118(2)
N(73^a)-H(73^a)...O11^a	1.00(4)	1.76(4)	2.726(4)	160(3)
O11^a-H11^a...Cl(2^a)#2	0.9583(10)	2.128(3)	3.085(2)	177(2)
O11^a-H21^a...O12^a	0.9583(10)	1.813(8)	2.743(4)	163(2)
O12^a-H12^a...Cl(2^a)	0.9585(10)	2.278(8)	3.196(3)	160.1(18)
O12^a-H22^a...O(48)#3	0.9585(10)	1.914(7)	2.858(3)	168(3)
O13^b-H13^b...Cl(2B^b)	0.9584(12)	2.24(9)	3.13(2)	154(18)
O13^b-H23^b...N(73B^b)	0.9584(11)	2.3(2)	2.82(2)	113(15)

Symmetry transformations used to generate equivalent atoms: #1 -x+1,-y+1,-z+2 #2 -x+1,-y,-z #3 x,y,z-1

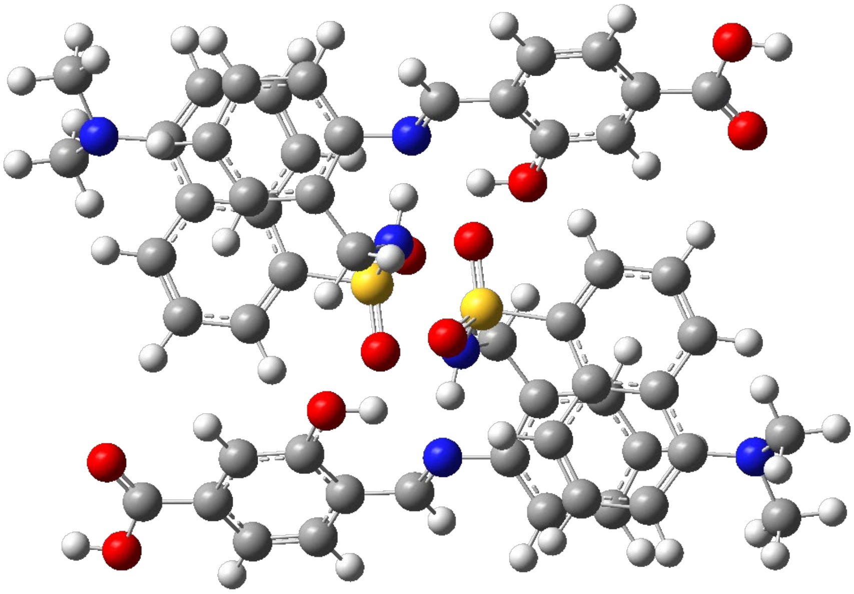

Figure S3. M062x/6-31G* optimized structure for the H_3L dimer.

Table S5. Selected bond lengths [Å], bond angles and dihedrals [°] for the $[H_4L]Cl\cdot0.92H_2O$ crystal and the theoretical geometry of the H_3L dimer

	**$[H_4L]Cl$ crystal structure**	**H_3L dimer M062X/6-31G***		**$[H_4L]Cl$ crystal structure**	**H_3L dimer M062X/6-31G***
O(1)-C(1)	1.341(3)	1.3379	O(41)-C(41)	1.342(3)	1.3376
C(7)-O(8)	1.196(3)	1.2053	C(47)-O(48)	1.215(3)	1.2093
C(7)-O(9)	1.314(3)	1.3474	C(47)-O(49)	1.327(3)	1.3454
C(10)-N(11)	1.282(3)	1.2836	C(50)-N(51)	1.286(3)	1.2801
N(11)-C(12)	1.420(3)	1.4124	N(51)-C(52)	1.423(3)	1.4132
C(18)-N(19)	1.479(3)	1.4640	C(58)-N(59)	1.484(3)	1.4810
N(19)-S(20)	1.6116(18)	1.6571	N(59)-S(60)	1.6080(19)	1.6585
S(20)-O(22)	1.4375(16)	1.4526	S(60)-O(61)	1.4339(16)	1.4579
S(20)-O(21)	1.4385(16)	1.4531	S(60)-O(62)	1.4388(16)	1.4556
S(20)-C(23)	1.773(2)	1.7926	S(60)-C(63)	1.791(5)	1.7903
C(28)-N(33)	1.483(3)	1.4204	C(68)-N(73)	1.485(4)	1.4194
N(33)-C(35)	1.493(3)	1.4620	N(73)-C(74)	1.491(4)	1.4506
N(33)-C(34)	1.497(3)	1.4516	N(73)-C(75)	1.505(4)	1.4612
O(1)-C(1)-C(2)	122.2(2)	123.10	O(41)-C(41)-C(42)	122.6(2)	123.17
O(8)-C(7)-O(9)	123.6(2)	122.23	O(48)-C(47)-O(49)	123.1(2)	122.21
C(10)-N(11)-C(12)	123.46(19)	120.26	C(50)-N(51)-C(52)	121.9(2)	120.20
N(19)-C(18)-C(17)	111.27(16)	110.88	N(59)-C(58)-C(57)	112.60(17)	110.90
C(18)-N(19)-S(20)	121.09(15)	119.13	C(58)-N(59)-S(60)	120.65(16)	119.20
O(22)-S(20)-O(21)	117.74(10)	120.95	O(61)-S(60)-O(62)	117.94(10)	120.88
N(19)-S(20)-C(23)	106.47(10)	106.05	N(59)-S(60)-C(63)	107.4(3)	106.06
C(28)-N(33)-C(35)	112.9(2)	113.62	C(68)-N(73)-C(74)	114.8(3)	115.64
C(28)-N(33)-C(34)	113.8(2)	115.64	C(68)-N(73)-C(75)	110.6(3)	113.59
C(35)-N(33)-C(34)	110.5(2)	110.80	C(74)-N(73)-C(75)	110.5(3)	110.84
C(10)-N(11)-C(12)-C(13)	15.58	-40.07			
C(18)-N(19)-S(20)-C(23)	-47.85(18)	-49.48			
C(71)-N(72)-C(73)-C(74)	15.58	-40.07			
C(58)-N(59)-S(60)-C(63)	52.0(2)	49.52			

Table S6. Hydrogen bonds for DsA [Å and deg.]

D-H···A	d(D-H)	d(H···A)	d(D···A)	<(DHA)
C(18)-H(17A)...O(22)[#1]	0.99	2.38	3.343(2)	163.9
C(34)-H(34B)...O(22)[#2]	0.98	2.47	3.231(2)	134.6
N(19)-H(19A)...O(21)[#3]	0.89(3)	2.15(3)	2.974(2)	154(2)
N(11)-H(11B)...O(21)[#4]	0.88(3)	2.32(3)	3.143(2)	156(3)
C(18)-H(17A)...O(22)[#1]	0.99	2.38	3.343(2)	163.9
C(34)-H(34B)...O(22)[#2]	0.98	2.47	3.231(2)	134.6
N(19)-H(19A)...O(21)[#3]	0.89(3)	2.15(3)	2.974(2)	154(2)
N(11)-H(11B)...O(21)[#4]	0.88(3)	2.32(3)	3.143(2)	156(3)

Symmetry transformations used to generate equivalent atoms: #1 -x+1/2, -y+3/2, -z+1 #2 x, -y+1, z-1/2 #3 -x+1/2, -y+1/2, z+1 #4 x+1/2, y+1/2, z

Conformer (Relative energy in kcal/mol) **Conformer (Relative energy in kcal/mol)**

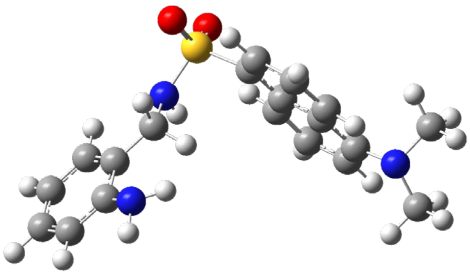

I

(1.50)

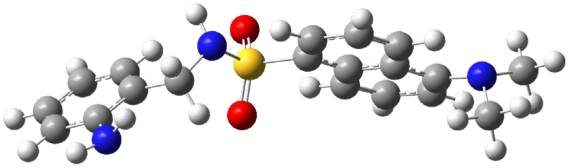

II

(3.20)

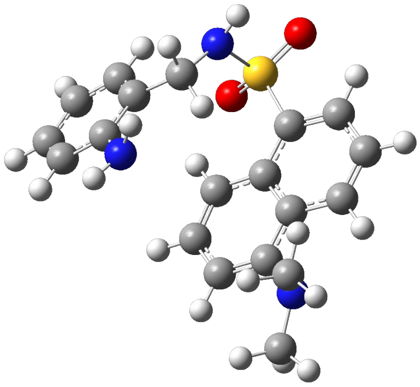

III

(2.46)

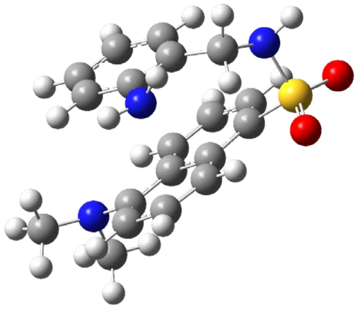

IV

(0.00)

Figure S4. M062X/6-31G* stable conformers for DsA. Relative energies (between brackets) are in kcal/mol. Since DsA has a certain conformational variability due to rotation around the $-CH_2-NH-SO_2-$ unit, we have characterised four stable conformers (I, II, III and IV). It must be noted that the conformer IV is even more stable than I, probably due to the stabilizing π-stacking interaction between parallel aromatic rings. These results suggest that the preference for the crystal structure I obtained experimentally is probably related to the stabilization due to hydrogen bonding between adjacent molecules in the solid phase.

Table S7. Selected bond lengths [Å], bond angles and dihedrals [°] measured and calculated for DsA

	**DsA crystal structure**	**DsA M062X/6-31G***
N(11)-C(12)	1.379(3)	1.3993
C(18)-N(19)	1.459(2)	1.4608
N(19)-S(20)	1.6135(17)	1.6666
S(20)-O(22)	1.4318(14)	1.4526
S(20)-O(21)	1.4427(14)	1.4512
S(20)-C(23)	1.7775(19)	1.7913
C(28)-N(33)	1.420(2)	1.4176
N(33)-C(35)	1.463(2)	1.4630
N(33)-C(34)	1.453(2)	1.4518
N(19)-C(18)-C(17)	113.01(16)	111.77
C(18)-N(19)-S(20)	120.30(14)	117.06
O(22)-S(20)-O(21)	118.47(8)	121.66
N(19)-S(20)-C(23)	108.22(9)	104.69
C(28)-N(33)-C(35)	114.86(15)	113.41
C(28)-N(33)-C(34)	115.10(16)	115.74
C(35)-N(33)-C(34)	110.49(16)	110.67
C(18)-N(19)-S(20)-C(23)	64.68(16)	56.53

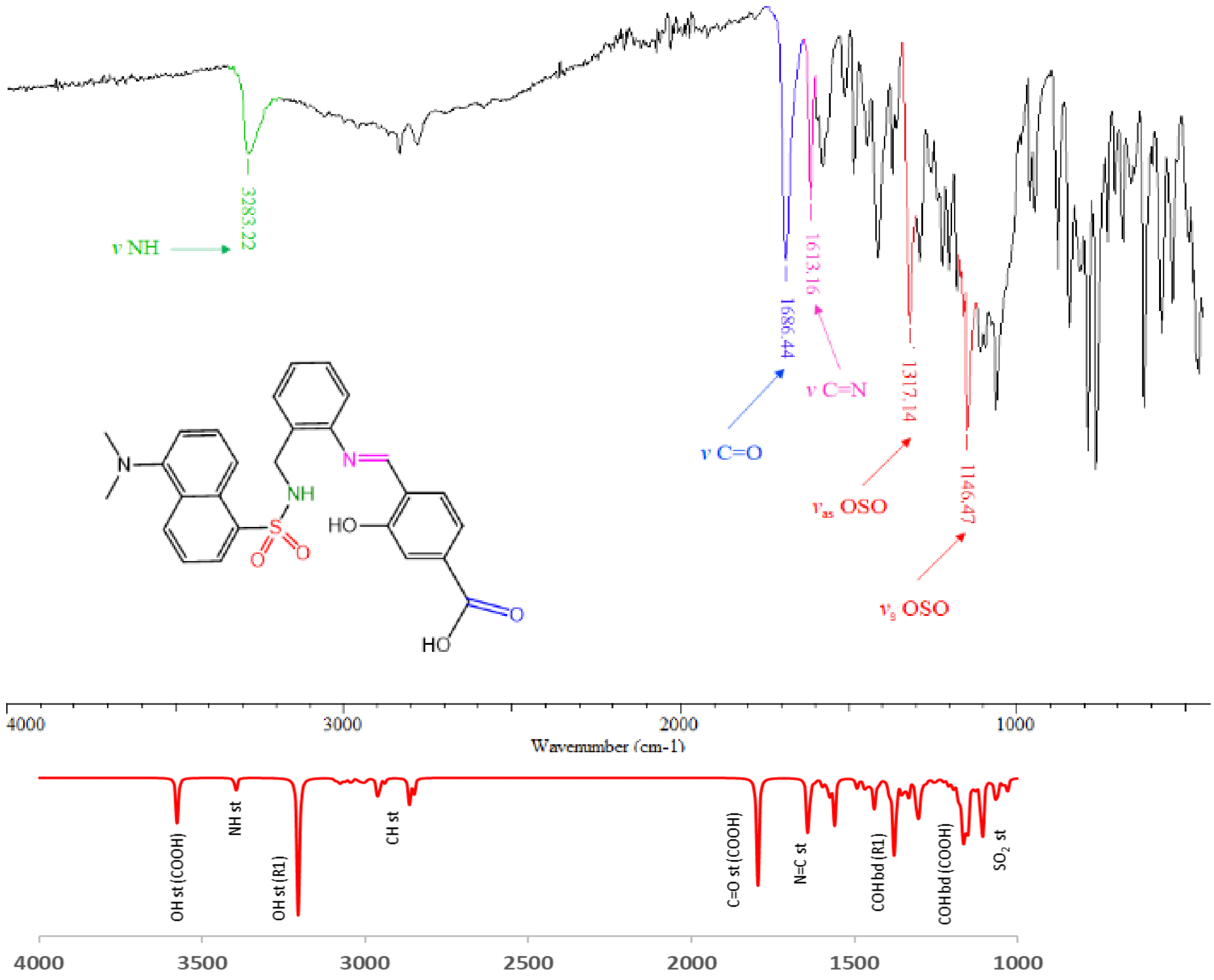

Figure S5. Experimental (top, ATR-FTIR) and calculated (bottom) IR spectrum of H_3L. Signal assignment of remarkable functional groups of the molecule has been included.

Table S8. Experimental and theoretical vibrational frequencies (cm^{-1}) for H_3L

Experimental IR	Theory	Normal vibrational mode assignment[a]
1052	1063	N-C stretch (aniline)
1145	1109	O=S=O symm stretch
1221	1164	C-O-H bending (COOH group)
1317	1303	O=S=O asymm stretch + N-H bending + CH_2 bending
1412	1377	C-O-H bending (phenolic ring)
1483	1441	C=C asymm stretch (phenolic ring) + C-O-H bend (phenolic ring)
1576	1562	C=C asymm stretch (phenolic ring) + C-O-H bend (phenolic ring)
1613	1642	N=C stretch
1686	1796	C=O stretch (COOH group)
2781	2850	C-H symm stretch (dansyl N-CH_3)
	2864	C-H symm stretch (dansyl N-CH_3)
2833	2957	C-H asymm stretch (dansyl N-CH_3)
	2960	C-H stretch (N=C-H unit)
	2962	C-H asymm stretch (dansyl N-CH_3)
3283	3204	O-H stretch (phenolic ring)
	3396	N-H stretch (aniline)
	3575	O-H stretch (COOH group)

[a] The unsigned mean error for theses assignments is 43 cm^{-1}.

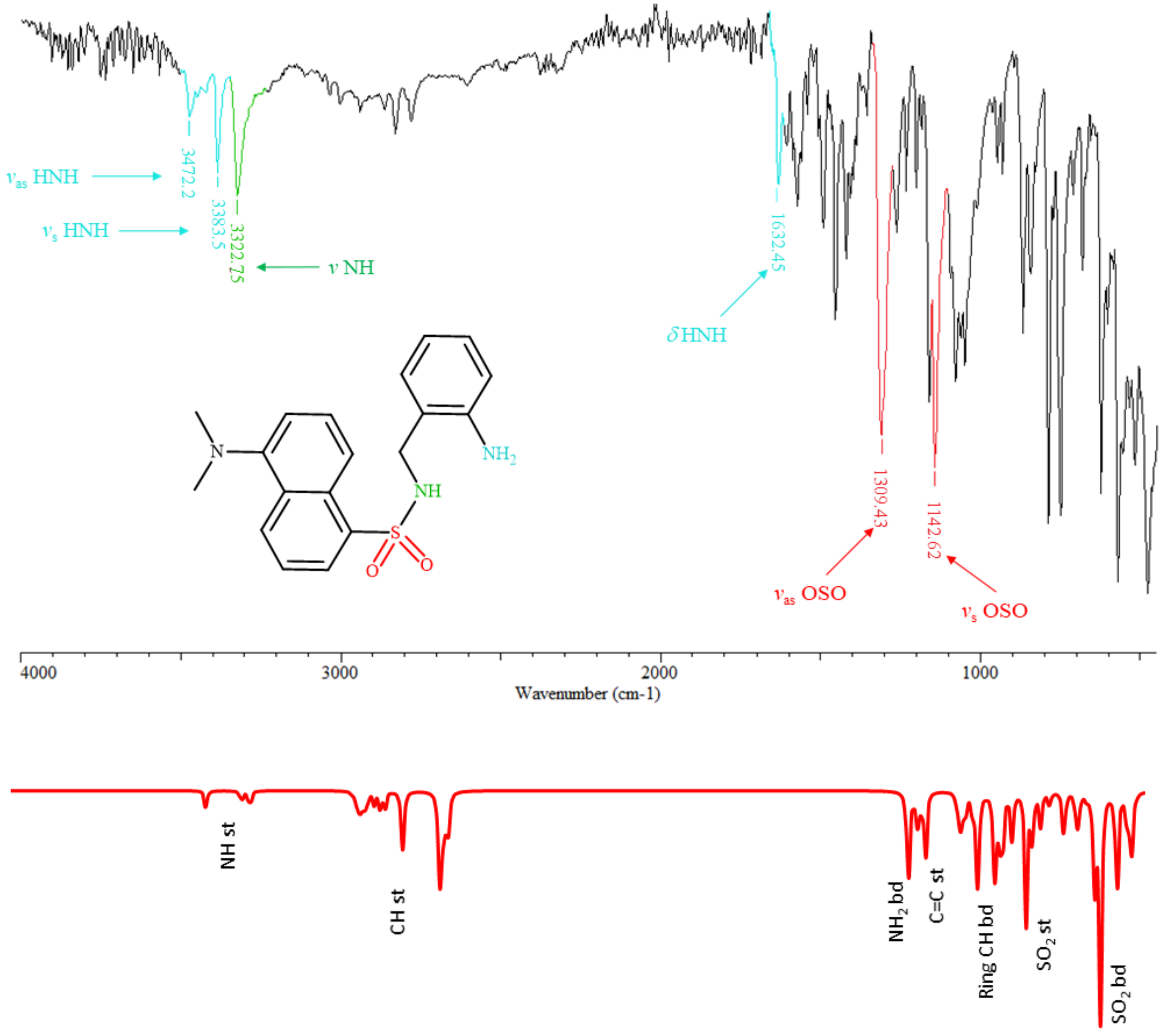

Figure S6. Experimental (top, ATR-FTIR) and theoretical profile (bottom) for the infrared spectrum of DsA. Signal assignment of remarkable functional groups of the molecule has been included.

Table S9. Experimental and theoretical vibrational frequencies (cm^{-1}) for DsA

Experimental	Theory	Normal vibrational mode assignment[a]
1048	1031	N-CH_3 bending (dansyl) + ring breathing (dansyl)
1076	1071	N-C stretch (aniline) + NCH_3 rocking (dansyl)
1142	1113	O=S=O symm stretch + ring C-H bending (aniline + dansyl)
1159	1130	Ring C-H bending (aniline)
1200	1173	Ring C-H bending (aniline)
1231	1212	Ring C-H bending (aniline)
1261	1272	Ring C-H bending (aniline)
	1296	Ring C-H bending (dansyl)
1310	1310	N-H bending (aniline) + CH_2 twist (aniline)+ O=S=O asymm stretch
1354	1346	C=C asymm stretch (dansyl)
	1349	CH_2 wagging (aniline)
	1375	N-H bending (aniline) + ring C-H bending (dansyl)
1420	1395	N-H bending (aniline) + ring C-H bending (dansyl)
1452	1438	Ring C-H bending (aniline) + ring C-H bending (dansyl)
	1440	Ring C-H bending (dansyl) + ring C-H bending (aniline)
1493	1482	Ring C-H bending (aniline)
1572	1578	C=C asymm stretch (dansyl)
	1601	NH_2 bending (aniline) + C=C asymm stretch (aniline)
1630	1624	NH_2 bending (aniline) + C=C asymm stretch (aniline)
2779	2840	CH_3 symm stretch (aniline –CH_2–)
	2853	CH_3 symm stretch (dansyl N-CH_3)
2830	2865	CH_3 symm stretch (dansyl N-CH_3)
	2913	CH_2 asymm stretch (aniline)
	2961	CH_3 asymm stretch (dansyl N-CH_3)
2940	2964	CH_3 asymm stretch (dansyl N-CH_3)
	3066	Ring C-H asymm stretch (aniline)
3035	3078	Ring C-H symm stretch (aniline)
3323	3367	N-H stretch (aniline)
3383	3389	NH_2 symm stretch (aniline)
3470	3484	NH_2 asymm stretch (aniline)

[a] The unsigned mean error for theses assignments is 21 cm^{-1}.

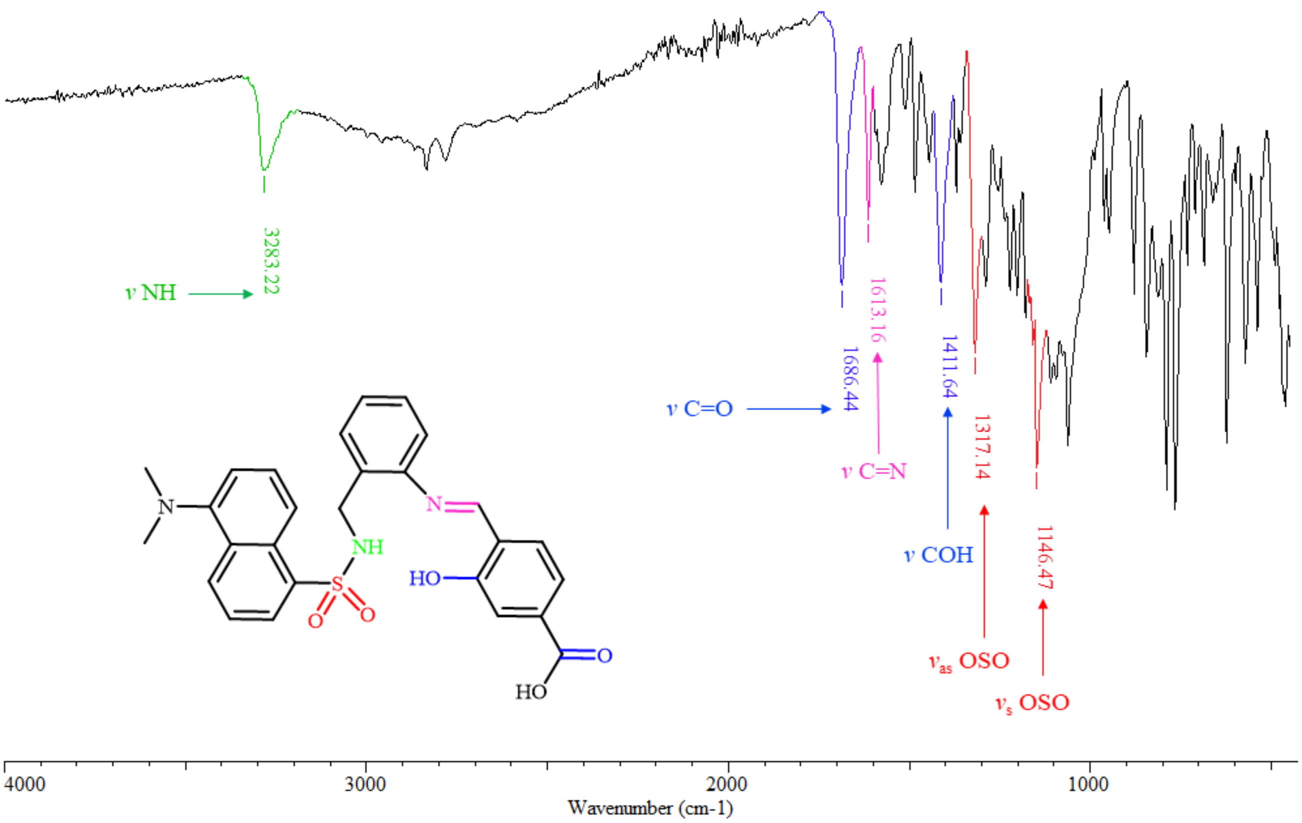

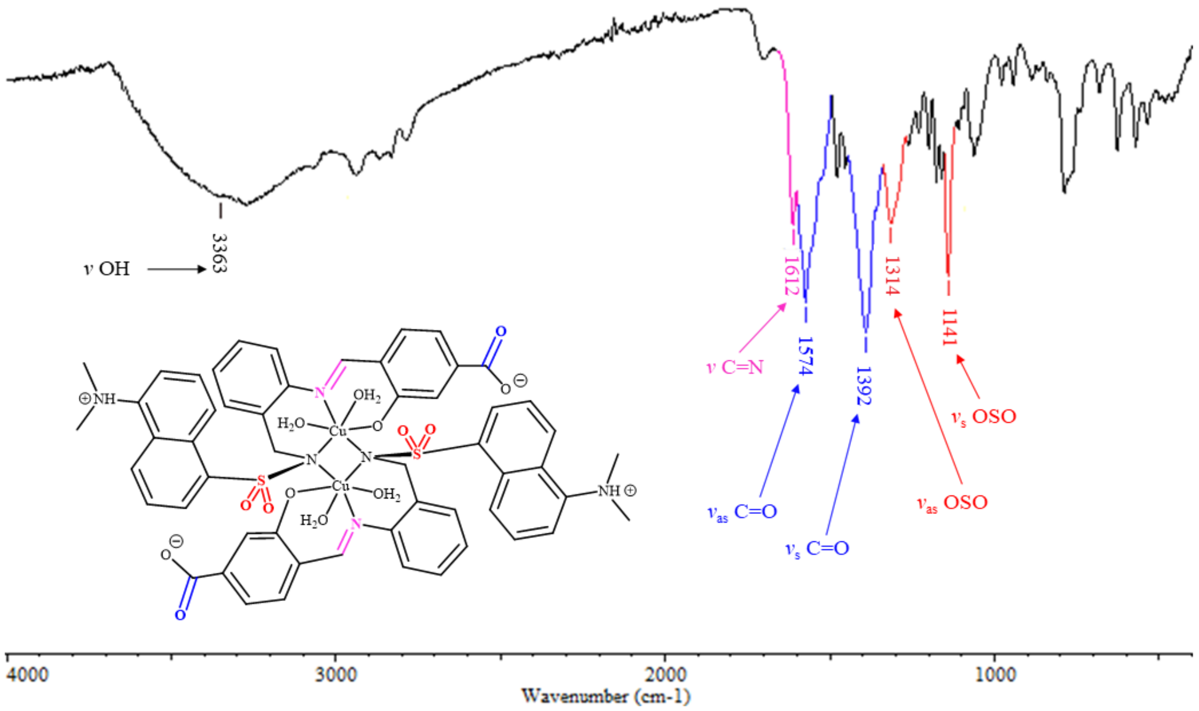

Figure S7. View of the ATR-FTIR spectra of the free ligand (top) and the copper(II) complex (bottom), with signal assignment of remarkable functional groups of the molecules on the corresponding schemes.

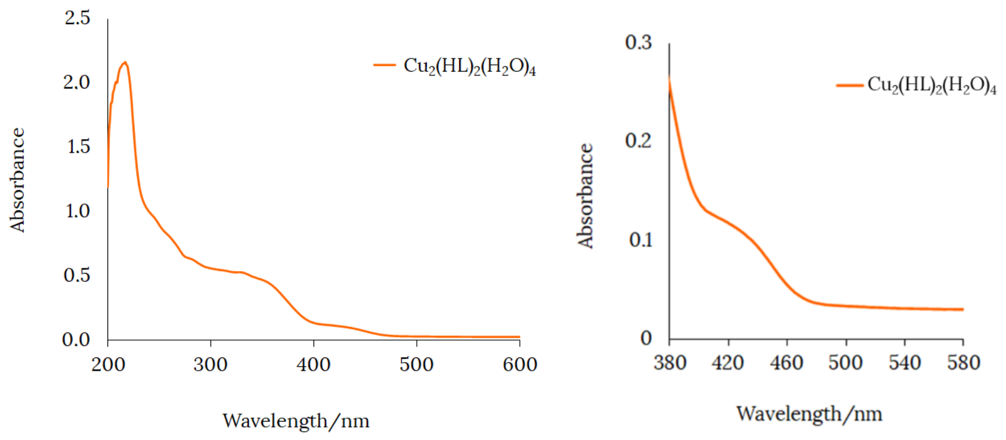

Figure S8. Left: View of the UV-Vis spectrum of the copper(II) complex in ethanol. Right: Magnification of spectrum in the region 380-580 nm to more easily see a new absorption band (as a shoulder) at about 410 nm (LMTC).

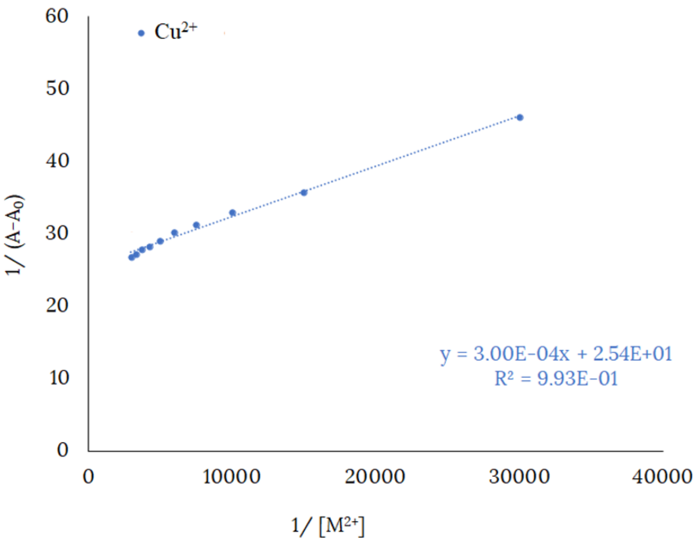

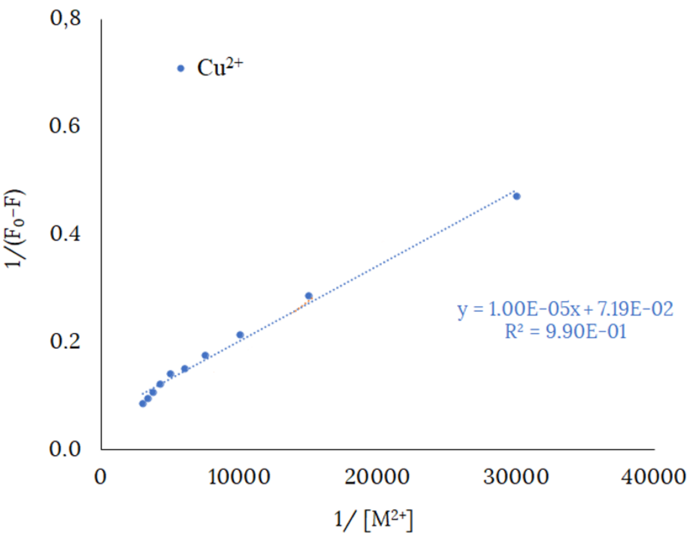

$$\frac{1}{A-A_0}=\frac{1}{A'-A_0}+\frac{1}{K(A'-A_0)[M^{2+}]}$$

$$\frac{1}{F-F_0}=\frac{1}{K\ (F_{max}-F_0)[M^{n+}]}+\frac{1}{F_{max}-F_0}$$

Kb	$[H_3L]$ = 100 µM
	$[M^{2+}]$ = 1000 µM

Solution Nº	1	2	3	4	5	6	7	8	9	10	11
H_3L/mL	1	1	1	1	1	1	1	1	1	1	1
M^{2+}/mL	0	0.1	0.2	0.3	0.4	0.5	0.6	0.7	0.8	0.9	1
$[M^{2+}]$/µM	0	33.3	66.7	100	133.3	166.7	200	233.3	266.7	300	333.3

Figure S9. Benesi–Hildebrand plot from UV-Vis (left) and fluorescence (right) titration data of H_3L (10^{-4} M) with Cu^{2+} (10^{-3} M) in an 80:20 ethanol:water solution.

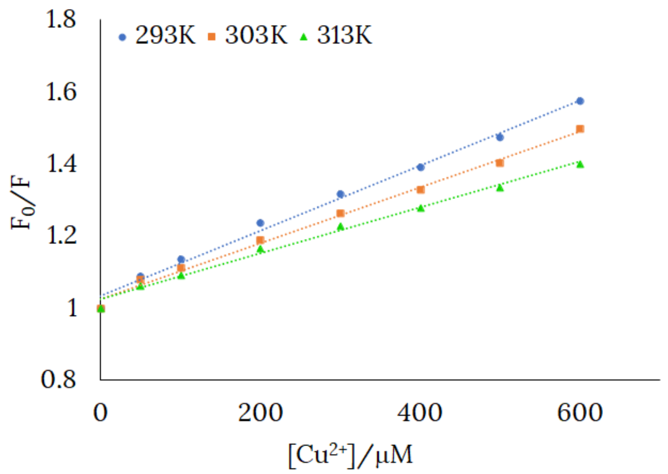

$$\frac{F_0}{F} = 1 + K_{sv}[Q]$$

Ksv	$[H_3L]$ = 100 μM
	$[M^{2+}]$ = 1000 μM

Solution N°	1	2	3	4	5	6	7	8
H_3L/mL	1	1	1	1	1	1	1	1
Quencher/mL	0	0.15	0.30	0.60	0.90	1.20	1.50	1.80
[Quencher]/μM	0	50	100	200	300	400	500	600

Figure S10. Plots of the intensities of the fluorescence spectra of H_3L (10^{-4} M) *vs* the concentration of the quenchers Cu^{2+} in an 80:20 ethanol:water solution. Slopes of the curves at 293, 303 and 313K are Stern-Volmer constants K_{SV} ($K_{SV} = k_q\tau$, where τ is the lifetime of the fluorophore) at the cited temperatures.

	1CuO	7CuO	32CuO
Monoanionic	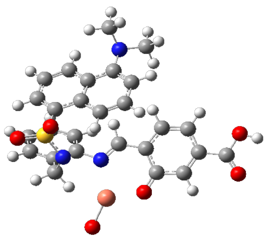 -61.2	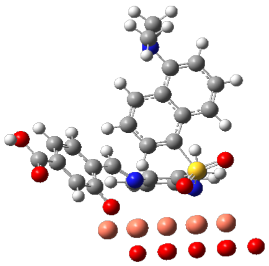 -145.3	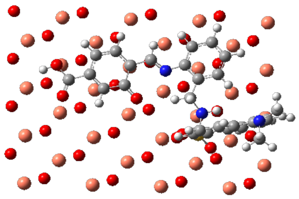 -170.0
Dianionic	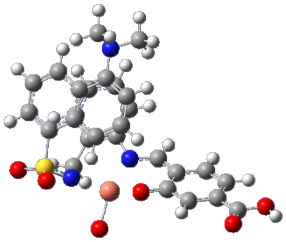 -76.6	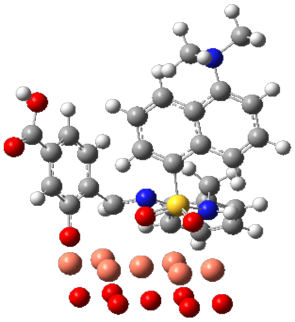 -189.5	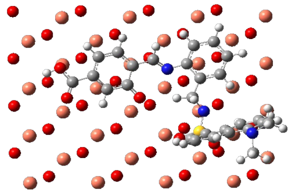 -273.4

Figure S11. Geometrical structures and interaction energies (in kcal/mol) for H_3L-CuO NPs interactions.
